# Supplementary material for: Biofertilizers from wastewater treatment as a potential source of mineral nutrients for growth of amaranth plants
Source: PLoS One. 2023 Dec 20;18(12):e0295624. doi: 10.1371/journal.pone.0295624 (PMC10732379; doi:10.1371/journal.pone.0295624)
Supplement: S1 Table — (PDF) [file pone.0295624.s001.pdf]

**S1 Table. Physical-Chemical characteristic of digested sewage.** Characteristics of anaerobically digested sewage by UAF (Upflow anaerobic filter) and BAR (baffled anaerobic reactor). Values are mean±SD of 72 and 24 replications for UAF and BAR, respectively. $P$, probability; TSS, total suspended solids; $DQO_d$, dissolved chemical oxygen demand.

| Parameters (mg $L^{-1}$) | UAF | BAR | Probability |
|---|---|---|---|
| Dissolved oxygen | $3.9 \pm 1.5$ | - | $P = 0.004$ |
| pH | $7.5 \pm 0.5$ | $7.5 \pm 0.5$ | $P = 0.623$ |
| TSS | $265.3 \pm 109.9$ | $211.1 \pm 111.5$ | $P = 0.063$ |
| $DQO_d$ | $178.6 \pm 50.1$ | $151.0 \pm 29.7$ | $P = 0.197$ |
| N-organic | $21.1 \pm 2.1$ | $24.4 \pm 10.6$ | $P = 0.820$ |
| N-ammoniacal | $90.8 \pm 20.1$ | $71.8 \pm 15.2$ | $P = 0.194$ |
| $NO_2$ | $0.4 \pm 0.6$ | $0.2 \pm 0.3$ | $P = 0.383$ |
| $NO_3$ | $12.4 \pm 7.9$ | $12.8 \pm 7.4$ | $P = 0.544$ |
| Total phosphorus | $11.3 \pm 2.0$ | $9.9 \pm 2.0$ | $P = 0.016$ |
| Phosphate | $27.0 \pm 5.5$ | $25.2 \pm 4.8$ | $P = 0.527$ |
| Alkalinity | $195.9 \pm 15.1$ | $196.2 \pm 23.9$ | $P = 0.312$ |
| Turbidity (NTU) | $220.3 \pm 64.1$ | $207.0 \pm 91.7$ | $P = 0.038$ |
