# Supplementary material for: Biofertilizers from wastewater treatment as a potential source of mineral nutrients for growth of amaranth plants
Source: PLoS One. 2023 Dec 20;18(12):e0295624. doi: 10.1371/journal.pone.0295624 (PMC10732379; doi:10.1371/journal.pone.0295624)
Supplement: S2 Table — (PDF) [file pone.0295624.s002.pdf]

S2 Table. Heavy metal concentration of digested sewage. Concentration of heavy metals of digested sewage after UAF (Upflow anaerobic Filter) and after BAR (baffled anaerobic reactor).

Parameters (mg L^{-1})	UAF	BAR
Al	0.635	0.402
As	0.465	0.110
Ba	0.180	0.121
Ca	17.984	15.347
Cd	0.013	0.006
Co	0.020	0.009
Cr	0.019	0.006
Cu	0.060	0.018
Fe	0.238	0.276
K	27.889	5.562
Mg	10.408	3.884
Mn	0.134	0.065
Na	26.140	48.773
Ni	0.033	0.010
Pb	0.039	0.070
Se	2.230	0.148
Zn	0.307	0.032
