# Supplementary material for: Biofertilizers from wastewater treatment as a potential source of mineral nutrients for growth of amaranth plants
Source: PLoS One. 2023 Dec 20;18(12):e0295624. doi: 10.1371/journal.pone.0295624 (PMC10732379; doi:10.1371/journal.pone.0295624)
Supplement: Attachment — Submitted filename: PONE-D-23-17844.pdf [file pone.0295624.s003.pdf]

PLOS ONE

Biofertilizers from wastewater treatment as a potential source of mineral nutrients for growth of amaranth plants

--Manuscript Draft--

**Manuscript Number:**	PONE-D-23-17844
**Article Type:**	Research Article
**Full Title:**	Biofertilizers from wastewater treatment as a potential source of mineral nutrients for growth of amaranth plants
**Short Title:**	Biofertilizers derived from treated domestic wastewater leads to sustainable agricultural practices
**Corresponding Author:**	Inês Cechin, Ph.D.<br>UNESP: Universidade Estadual Paulista Julio de Mesquita Filho<br>BAURU, SP BRAZIL
**Keywords:**	Growth; Organic fertilizer; Photosynthesis; Plant nutrition; Resource recovery
**Abstract:**	Exploring alternative fertilizers is crucial in agriculture due to the cost and environmental impact of inorganic options. This study investigated the potential of biofertilizers derived from sewage treatment on the growth and physiology of Amaranthus cruentus plants. Various treatments were compared, including control treatments with inorganic fertilizer and treatments with biofertilizers composed of microalgae and biosolids. Results showed high concentrations of essential nutrients, mainly Zn, Fe and Na, in the dry microalgae and biosolids, and their application led to increased shoot biomass and improved photosynthetic rates as the doses were increased. Zn and Na increased in the leaf of plants treated with dry microalgae and biosolid. Additionally, reclaimed water obtained after tertiary treatment of sewage with microalgae and biosolids alone showed promising effects on plant growth. Overall, these findings suggest that organic fertilizers derived from sewage treatment have the potential to enhance plant growth and contribute to sustainable agricultural practices.
**Order of Authors:**	Inês Cechin, Ph.D.
	Elisa Teófilo Ferreira
	Sarah Corrêa Barrochelo
	Sarah de Paula de Melo
	Thainá Araujo
	Augusto Cesar Coelho Xavier
	Gustavo Henrique Ribeiro da Silva
**Additional Information:**	
**Question**	**Response**
**Financial Disclosure**<br><br>Enter a financial disclosure statement that describes the sources of funding for the work included in this submission. Review the submission guidelines for detailed requirements. View published research articles from *PLOS ONE* for specific examples.<br><br>This statement is required for submission and **will appear in the published article** if the submission is accepted. Please make	The funders had no role in the study design.

Powered by Editorial Manager® and ProduXion Manager® from Aries Systems Corporation

sure it is accurate.<br><br>**Unfunded studies**<br>Enter: *The author(s) received no specific funding for this work.*<br><br>**Funded studies**<br>Enter a statement with the following details:<br>• Initials of the authors who received each award<br>• Grant numbers awarded to each author<br>• The full name of each funder<br>• URL of each funder website<br>• Did the sponsors or funders play any role in the study design, data collection and analysis, decision to publish, or preparation of the manuscript?<br>• **NO** - Include this sentence at the end of your statement: *The funders had no role in study design, data collection and analysis, decision to publish, or preparation of the manuscript.*<br>• **YES** - Specify the role(s) played.<br><br>* typeset	
**Competing Interests**<br><br>Use the instructions below to enter a competing interest statement for this submission. On behalf of all authors, disclose any competing interests that could be perceived to bias this work—acknowledging all financial support and any other relevant financial or non-financial competing interests.<br><br>This statement is required for submission and **will appear in the published article** if the submission is accepted. Please make sure it is accurate and that any funding sources listed in your Funding Information later in the submission form are also declared in your Financial Disclosure statement.<br><br>View published research articles from *PLOS ONE* for specific examples.	The authors have declared that no competing interests exist.

Powered by Editorial Manager® and ProduXion Manager® from Aries Systems Corporation

**NO authors have competing interests**<br><br>Enter: *The authors have declared that no competing interests exist.*<br><br>**Authors with competing interests**<br><br>Enter competing interest details beginning with this statement:<br><br>*I have read the journal's policy and the authors of this manuscript have the following competing interests: [insert competing interests here]*<br><br>* typeset	
**Ethics Statement**<br><br>Enter an ethics statement for this submission. This statement is required if the study involved:<br><br>• Human participants<br>• Human specimens or tissue<br>• Vertebrate animals or cephalopods<br>• Vertebrate embryos or tissues<br>• Field research<br><br>Write "N/A" if the submission does not require an ethics statement.<br><br>General guidance is provided below. Consult the submission guidelines for detailed instructions. **Make sure that all information entered here is included in the Methods section of the manuscript.**	N/A

Powered by Editorial Manager® and ProduXion Manager® from Aries Systems Corporation

**Format for specific study types**<br><br>**Human Subject Research (involving human participants and/or tissue)**<br>• Give the name of the institutional review board or ethics committee that approved the study<br>• Include the approval number and/or a statement indicating approval of this research<br>• Indicate the form of consent obtained (written/oral) or the reason that consent was not obtained (e.g. the data were analyzed anonymously)<br><br>**Animal Research (involving vertebrate animals, embryos or tissues)**<br>• Provide the name of the Institutional Animal Care and Use Committee (IACUC) or other relevant ethics board that reviewed the study protocol, and indicate whether they approved this research or granted a formal waiver of ethical approval<br>• Include an approval number if one was obtained<br>• If the study involved *non-human primates*, add *additional details* about animal welfare and steps taken to ameliorate suffering<br>• If anesthesia, euthanasia, or any kind of animal sacrifice is part of the study, include briefly which substances and/or methods were applied<br><br>**Field Research**<br><br>Include the following details if this study involves the collection of plant, animal, or other materials from a natural setting:<br>• Field permit number<br>• Name of the institution or relevant body that granted permission	
**Data Availability**<br><br>Authors are required to make all data underlying the findings described fully available, without restriction, and from the time of publication. PLOS allows rare exceptions to address legal and ethical concerns. See the PLOS Data Policy and FAQ for detailed information.	Yes - all data are fully available without restriction

A Data Availability Statement describing where the data can be found is required at submission. Your answers to this question constitute the Data Availability Statement and **will be published in the article**, if accepted.<br><br>**Important:** Stating 'data available on request from the author' is not sufficient. If your data are only available upon request, select 'No' for the first question and explain your exceptional situation in the text box.<br><br>Do the authors confirm that all data underlying the findings described in their manuscript are fully available without restriction?	
**Describe where the data may be found in full sentences. If you are copying our sample text, replace any instances of XXX with the appropriate details.**<br><br>• If the data are **held or will be held in a public repository**, include URLs, accession numbers or DOIs. If this information will only be available after acceptance, indicate this by ticking the box below. For example: *All XXX files are available from the XXX database (accession number(s) XXX, XXX.*).<br>• If the data are all contained **within the manuscript and/or Supporting Information files**, enter the following: *All relevant data are within the manuscript and its Supporting Information files.*<br>• If neither of these applies but you are able to provide **details of access elsewhere**, with or without limitations, please do so. For example:<br><br>*Data cannot be shared publicly because of [XXX]. Data are available from the XXX Institutional Data Access / Ethics Committee (contact via XXX) for researchers who meet the criteria for access to confidential data.*<br><br>*The data underlying the results presented in the study are available from (include the name of the third party*	All data can be obtained from the corresponding author under request.

and contact information or URL).<br>• This text is appropriate if the data are owned by a third party and authors do not have permission to share the data.<br><br>* typeset	
Additional data availability information:	

Manuscript Click here to access/download;Manuscript;Plos_one_2023.pdf

Biofertilizers from wastewater treatment as a potential source of mineral nutrients for growth of amaranth plants

Elisa Teófilo Ferreira[1¤], Sarah Corrêa Barrochelo[1‡], Sarah de Paula de Melo[1‡], Thainá Araujo[1‡], Augusto Cesar Coelho Xavier[1‡], Inês Cechin[1◑*†], Gustavo Henrique Ribeiro da Silva[2¶]

1 Department of Biological Sciences, Faculty of Sciences, UNESP – São Paulo State University, Bauru, SP CEP 17033-360, Brazil
2 Department of Civil Engineering, Faculty of Engeneering, UNESP – São Paulo State University, Bauru, SP CEP 17033-360, Brazil

◑IC conceived the project, contributed to the writing of the manuscript, helped to conduct the experiments, and contributed to the data analysis.
¶GHRS conceived the project, provided valuable suggestions for the manuscript, and provided the biofertilizers used in the experiments.
¤ETF contributed to the writing of the manuscript, helped to conduct the experiments, and contributed to the data analysis.
†SCR, SPM, TA, and ACCX conducted the experiments.
* ines.cechin@unesp.br

Abstract

Exploring alternative fertilizers is crucial in agriculture due to the cost and environmental impact of inorganic options. This study investigated the potential of biofertilizers derived from sewage treatment on the growth and physiology of *Amaranthus cruentus* plants. Various treatments were compared, including control treatments with inorganic fertilizer and treatments with biofertilizers composed of microalgae and biosolids. Results showed high concentrations of essential nutrients, mainly Zn, Fe and Na, in the dry microalgae and biosolids, and their application led to increased shoot biomass and improved photosynthetic rates as the doses were increased. Zn and Na increased in the leaf of plants treated with dry microalgae and biosolid. Additionally, reclaimed water obtained after tertiary treatment of sewage with microalgae and biosolids alone showed promising effects on plant growth. Overall, these findings suggest that organic fertilizers derived from sewage treatment have the potential to enhance plant growth and contribute to sustainable agricultural practices.

Introduction

As the global population continues to grow, so does the generation of waste and demand for resources. Unfortunately, the linear production model that we are inserted in often leads to inefficient use of resources and waste generation [1]. The circular economy proposes a shift in production chains towards closed-loop processes to improve resource efficiency and reduce waste [2,3]. In this context, sewage is no longer considered as a waste, but as a valuable source for nutrient recovery [4,5], which can be used to restore soil fertility and organic matter. Soil fertilization and the replacement of organic matter is essential due to the depletion of nutrients from the soil over the consecutive seasons,

without which it is impossible to meet the food demand for a growing population [6]. As a result, exploring alternative sources of fertilizers is of great importance in agriculture due to the high cost and negative environmental impact of inorganic fertilizers. According to the literature, the resources recovered from the treatment of sanitary sewage could potentially meet up to 30% of the agricultural demand for N [7] and 22% of the demand for P [8, 9], thereby reducing the need for chemical fertilizers.

Sewage sludge is a residue rich in organic matter generated during the treatment of sewage in sewage treatment plants (STP). The characteristic of biological sewage sludge depends on the source of sewage which may contain heavy metals in addition to mineral nutrients and organic matter [10]. However, when subjected to additional treatments in order to reduce the water content and/or to stabilize the organic matter and remove pathogens [11, and references therein], it results in a biosolid that is easy to handle for disposal or use as a biofertilizer in agriculture because it has a large amount of nutrients and organic matter [12]. Several studies have been conducted in order to verify the benefits of biosolid application on several crops' growth and biochemical parameters [13–15]. In addition, the application of biosolid in areas undergoing reclamation can effectively improve the performance of introduced vegetation and the tolerance to potentially toxic elements [16] thus serving as a key tool for farm utilization [17].

Microalgae-based treatment of sewage is a promising method for nutrient recovery and wastewater treatment. After anaerobic treatment, the remaining effluent is favorable for the cultivation of microalgae as it retains most of the nutrients [18–20]. The ability of microalgae to use inorganic compounds such as nitrogen and phosphorus for their growth makes them efficient at removing nutrients from wastewater, which can reach up to 90% [21, 22]. Additionally, microalgae can help in disinfection by varying the pH and improving the quality of the final effluent [19, 23, 24]. The recovery of chemical energy and nutrients from microalgae biomass is also possible. According to [25], 68% of the chemical energy of microalgal biomass can be recovered, in the form of bio-oil (44%) and biogas (23%), in addition to the recovery of 44% of the N content and 91% of the P content of biomass in the form of fertilizer products. The organic matter of microalgae can also be used as a nutritive material with advantageous characteristics for crops and soil. From the organic matter of these microalgae, nutritive material can be obtained with advantageous characteristics for the crops and the soil, depending on the microalgae's ability to improve soil quality, its microflora and increase productivity [9, 26]. Studies have shown that organic microalgae fertilizer grown in wastewater can improve soil quality and increase crop productivity [5, 26, 27].

The reuse of treated domestic sewage in agriculture can provide multiple benefits. Reclaimed water is a valuable alternative to traditional irrigation water sources and can contribute to reducing the pressure on freshwater resources [28]. The tertiary treatment of sewage with microalgae does not remove 100% of nutrients of the reclaimed water. Therefore, this treated sewage constitutes another by-product that can be destined for agriculture. Fertigation of plants with reclaimed water has proved to be very advantageous in regions where water availability is low [29, 30]. In addition to its value as a source of water, reclaimed water has also been shown to be efficient in increasing the productivity of several crops of economic interest due to the presence of essential nutrients necessary for plant growth despite the presence of some metals [31–33].

The genus of *Amaranthus* comprises plants of great agronomic importance because the nutritional quality of leaves and seeds is related to large amounts of proteins, vitamins and minerals and having a better balance in amino acid composition than cereals such as wheat, corn, and sorghum [34] besides being gluten free, making it important for celiac patients [35]. Bearing in mind the importance of recycling the nutrients contained in the sewage, the present study examined the effectiveness of

here I would suggest to add review of already work done on similar aspects and what is needed to highlight the research gap plz

sewage treatment by-products as organic fertilizer i[illegible]nthus *cruentus* L. Specifically, the study analyzed the res[illegible]tary sewage treatment by-products in terms of growth, shoot bi[illegible]tosynthetic pigments, leaf gas exchange and leaf nutrients. It wa[illegible]e addition of sanitary sewage treatment by-products may positiv[illegible]mance of amaranth plants thus resulting in reduced fertilizer [illegible]ral resources and avoiding environmental pollution. The study c[illegible]to the potential benefits and limitations of this approach.

Materials and methods

Obtaining organic fertilizers

Collection of previously treated sewage and biosolid obtantion

The sanitary sewage previously anaerobically treated used for cultivation of microalgae was collected at the STP in the district of Tibiriçá, in Bauru/Sao Paulo (22°13’38.7”S 49°1 2’47.0”W). The anaerobic treatment took place in a anaerobic upflow filter, with a volume of 87.30 m^3 and a hydraulic detention time (HDT) of 5.05 h. The sludge was obtained in the third chamber of the baffled anaerobic reactor, which had three chambers with a total volum of 597 L and HDT of 8 h. After collecting the sludge was oven dried at 60°C. Then, it was crushed and ground in a mill to facilitate the absorption by the plants.

Microalgae cultivation

The microalgae were cultivated in a transparent box with 50 L of anaerobically pre-treated sewage with 5 L of inoculum of native microalgae from sanitary wastewater. The cultivation of microalgae took place at room temperature under a shading fabric that blocked 50% of natural light radiation (Solpack), and continuously air injection (10 L min^{-1}) by air compressors (Seven Star - S-6000) connected to microbubble hose (Aquadrop Air, Model M25), during seven days. After this period, the effluent was transferred to 200 L drums. The microalgae cultivation took place between the months of January and April (summer/fall), with an average temperature of 28^0C, and the average light intensity of 919.48 μmol m^{-2} s^{-1}. Eleven weeks of cultivation were necessary to obtain the amount of microalgae established for supplementation of the plants.

Sedimentation of microalgae, obtaining reclaimed water and biomass from microalgae

After filling the decantation drums with the microalgae together with the effluent, 100 $mg.L^{-1}$ of tannin (Tanfloc) were added for the coagulation, flocculation and sedimentation of the microalgae [36]. After 24 hours the addition of tannin, all the reclaimed water and the sedimented microalgae were collected.

Plant Material and Growth Conditions

Seeds of *Amaranthus cruentus* L. cv. BRS Alegria were used. This cultivar was developed by the 13th Cerrados Agricultural Research Center, originated from the strain *A. cruentus* AM 5189 from United States [37]. The experiment was carried out in the greenhouse at the Department of Biological Sciences at the Sao Paulo State University/Bauru Campus, under natural photoperiodic conditions and minimum and

maximum average temperature of 17 and 33°C, respectively. The seeds were sown in 4 L plastic pots filled with vermiculite. After one week, the plants were thinned to one per pot. Each pot received 250 mL of 20% or 70% full strength Long Ashton (LA) solution [38] from sowing and continued three times a week.

Dried and fresh microlgae application

After sedimentation, part of the microalgae was kept in a refrigerator before using and the other was distributed in plastic trays for drying in a forced air circulation oven at 65°C for 48 hours. The fresh and the dry microalgae were applied in two periods: 20 days before sowing and 24 days after sowing. The plants were divided into two main groups: (I) with or without dry microalgae or (II) with or without fresh microalgae. Each group was divided into five subgroups:

1 Group I: (1) 70% LA; (2) 20% LA; (3) 20% LA + 2 g of dry microalgae; (4) 20% LA + 4 g of dry microalgae and (5) 20% LA + 8 g of dry microalgae.

2 Group II: (1) 70% LA; (2) 20% LA; (3) 20% LA + 3 g of fresh microalgae; (4) 20% LA + 6 g of fresh microalgae and (5) 20% LA + 9 g of fresh microalgae.

Reclaimed water application

The reclaimed water obtained after separating the microalgae was kept in a refrigerator before using as a plant nutritive solution. The plants were divided into three groups: (1) 70% LA; (2) 20% LA and (3) reclaimed water. The reclaimed water was applied in the same volume as the Long Ashton solution, three times a week.

Biosolid application

Two biossolid applications were made: the first 20 days before sowing and the second about 20 days after sowing. The plants were divided into six groups: (1) 70% LA; (2) 20% LA; (3) 20% LA + 3 g of biosolid; (4) 20% LA + 6 g of biosolid; (5) 20% LA + 10 g of biosolid and (6) tap water + 10 g of biosolid.

Growth measurements

At approximately 7 day intervals, the height (cm), the diameter (mm) of the stem at insertion of cotyledons and the number of visible leaves were measured. A millimeter ruler was used for height measurements and a digital caliper (KingTools) was used for stem diameter measurements.

Gas exchange measurements and photosynthetic pigments determination

An infrared gas analyzer (LCpro, ADC, Hodde [illegible] r measurements of photosynthesis (A), stomatal conductance (g [illegible] d CO_2 concentration in the substomatic cavity (C_i), [illegible] xpanded leaf after 44 days of cultivation. The measurements wer [illegible] greenhouse between 8 am and 10 am under ambient cond [illegible] partial pressure of CO_2 and water vapour pressure of the air. Pho [illegible] adiation (PAR) of 1000 μmol m^{-2} s^{-1} was supplied by a lamp coupled on the top leaf chamber. The leaf was kept under this PAR until a steady-state rate was achieved. Total chlorophyll and carotenoids were extracted from leaf discs of known area in 80% aqueous acetone according to [39] and the concentration expressed on a leaf area basis (g m^{-2}).

Write exact instrument specification, including model number, comapny and city of manufacture etc

Dry Mass Determination

At the end of the experimental period, plants of each treatment were selected randomly for dry matter determinations. The plants were divided into stem and leaves before been oven dried at 60°C for 48 h and the dry matter was expressed as g plant^{-1}.

Chemical analysis: microalge, reclaimed water, biosolid and amaranth leaves

The analyses were done in the Laboratory of Soil, located in the Department of Soils and Environmental Resources at the Faculty of Agricultural Sciences/UNESP/Botucatu/SP. Chemical leaf nutrients status was determined in oven dried and finely ground leaf by the semi-micro-Kjeldahl method, after sulphurous digestion according to [40]. The determination of macronutrients (N, P, K, Ca, Mg and S) and micronutrients (Na, Cu, Fe, Mn, Zn and Na) of dry and wet microalgae, biosolid and reclaimed water was performed according to the methodology of the Manual of Official Analytical Methods for Fertilizers and Correctives. Metal analysis (Co, Cr, Ni, Pb and Se) of the biosolid was performed according to [41].

Statistical analysis

Each plants group has been analyzed separately plz mentions here

The data were submitted to simple a[illegible]OVA) by using the software SPSS/PC 9.0 for Windows. Quantit[illegible]ferent variables were analyzed using a test of multiple con[illegible]differences between treatments at 5% significance level. T[illegible]ference (LSD) test was used for assumed equal variances and the Games-Howell test for non-assumed equal variances. The statistical analyzes for the number of leaves were performed after transforming the data into $\sqrt{x + 0.5}$.

Results

Growth and dry matter production

In this study, four different biofertilizers from the treatment of sewage were applied in plants grown under 20% full strength LA solution in order to verify the potential of these biofertilezer in supplying nutrients for growth of amaranth plants (Fig. 1). In well nourish plants (70% LA), the plant height, stem diameter and number of leaves were significantly higher than under lower strength nutrient solution (20% LA) at the end of experimental period. From 42 days after sowing, the number of leaves produced in the plants grown under 70% LA practically remained constant until the end of the experiment due to the beginning of flowering. Comparing the different applications of dry microlagae with 20% LA after 28 days of sowing, it can be seen that the plants supplied with 4 g of microalgae presented significantly lower height while the other did not differ (Fig. 1A). From 35 days after sowing and until the end of the experiment, the plants supplied with 4 g and 8 g dry microalgae presented similar values in height, reaching at the end of experiment 48.5 and 51.5 cm, respectively, significantly surpassing 20% LA, which reached 40.3 cm, and the 2 g dry microalgae which reached 39.6 cm (Fig. 1A). The number of leaves produced per plant began to differ significantly between treatments after 28 days of sowing (Fig. 1B). The plants grown under 8 g of dry microalgae reached the number of leaves produced in 70% LA 49 days after sowing, with both treatments presenting an average of 24 leaves per plant (Fig. 1B). After 56 days of sowing, the number of leaves in 8 g of dry microalgae exceeded the number of leaves of 70% LA, reaching 26 leaves per plant. The stem diameter of the plants grown

under supplementation with dry microalgae at the beginning of the experiment were very similar to the stem diameter of the 20% LA. At the end of experiment, the stem diameter of the of 4 g and 8 g of dry microalgae showed similar values, 8.1 and 8.7 mm, respectively, values significantly higher than the stem diameter of 20% LA and 2 g of dry microalgae (Fig. 1C).

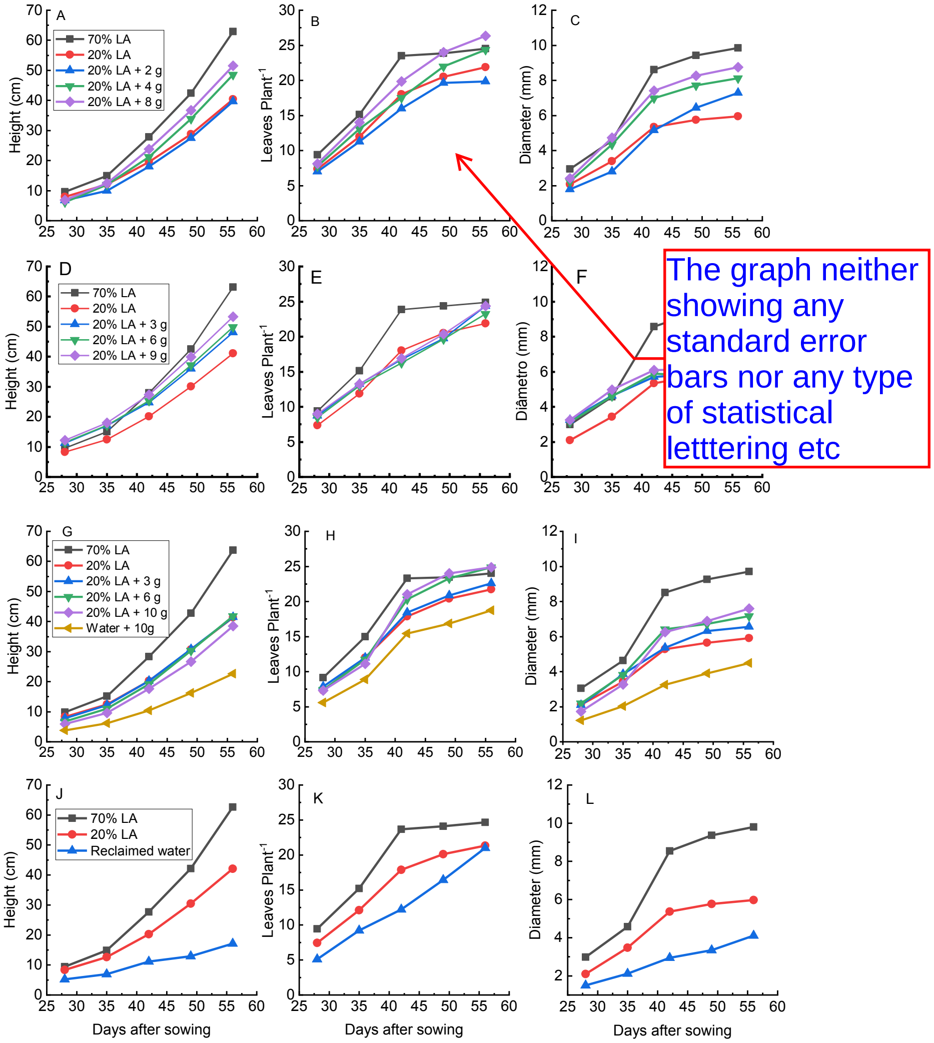

Fig 1. Plant height (A, D, G and J), number of leaf per plant (B, E, H and K) and stem diameter (C, F, I and L) of amaranth plants grown under addition of dry microalgae (A, B and C), wet microalgae (D, E, and F), biosolid (G, H and I) or reclaimed water (J, K and L) during the growth period. Values are mean of 8 plants.

In Figure 1D, it is noted that in the first two weeks of measurements the plants supplied with wet microalgae (3 g, 6 g and 9 g) presented heights greater than the two controls (20% and 70% LA). From 42 days after sowing, the treatments with wet microalgae resulted in heights greater than the treatment with 20% LA, but lower than the treatment with 70% LA, with the treatment with 9 g of wet microalgae showing the highest height among the treatments with wet microalgae. Regarding the number of leaves produced, the treatments with wet microalgae, between 28 and 35 days after sowing and at the end of the experiment, showed a slightly higher number than 20% LA (Fig. 1E). At the end of the experiment, the number of leaves in plants grown with the

addition of 3 g and 9 g of wet microalgae practically equalled that of 70% LA. The diameter of stem in plants grown under addition of wet microalgae had values higher than the 20% LA control and similar values to the control 70% LA until about 35 days after planting (Fig. 1F). After this period, the treatments with wet microalgae were not significantly different from 20% LA.

Plants grown under 3 g and 6 g of biosolid plus 20% LA did not differ significantly from the 20% LA control in terms of height of the plants over the period of growth (Fig. 1G). However, the supply of 10 g of biosolid with 20% LA or with 10 g of pure biosolid showed a reduction of 10% and 47% at the end of growth period in relation to the 20% control, respectively. Between 28 and 35 days after sowing, the number of leaves per plant did not differ significantly between the control 20% LA and the doses of 3 g, 6 g and 10 g of biosolid plus 20% LA (Fig. 1H). However, plants grown with only 10 g of biosolid showed lower values than the other treatments. At the end of the experiment, plants with 6 g and 10 g of biosolid plus 20% LA had significantly higher number of leaves than the 20% LA control and 3 g of biosolid plus 20% LA and the pure biosolid, but equal to the 70% control. After 28 days of sowing, the application of biosolid at doses of 3 g and 6 g per pot did not result in a significant change in stem diameter compared to 20% LA (Fig. 1I). However, application of 10 g of biosolid with 20% LA or 10 g of biosolid with tap water resulted in a significant reduction in stem diameter of 18% and 42%, respectively. At the end of the experiment, there was a positive correlation between the biosolid doses applied together with 20% LA and the stem diameter. However, the application of biosolid with tap water presented a reduction of approximately 24% in the stem diameter compared to 20% LA.

At 28 days after sowing, the height of plants treated only with reclaimed water reached approximately 62% and 55% of the two controls 20% LA and 70% LA, respectively. At the end of the experimental period, the height of plants under reclaimed water presented much lower percentages (about 42% and 27%, respectively). At the beginning of the experiment, the average number of leaves in the reuse water was 5.1, representing about 69% and 54% of the controls 20% LA and 70% LA, respectively (Fig. 1K). Despite the reclaimed water having initially affected the production of leaves, at the end of the experiment the production was equal to the control 20% LA. This was due to the fact that the plants under 20% stopped producing new leaves because they entered the reproductive phase earlier than the plants in the reclaimed water. The diameter of stem 28 days after sowing represented 72% and 50% of the two controls, respectively (Fig. 1L). At the end of experimental period, the diameter of stem of the plants treated only with reclaimed water reached 69% and 42% of the two controls 20% LA and 70% LA, respectively.

Dry mass production in the shoot under 4 g and 8 g of dry microlagae significantly exceeded the 2 g and 20% LA (Fig. 2A). The plants under 8 g of dry microalgae produced more than twice the dry mass produced in 20% LA, and produced only 30% less compared to 70% LA. The dry microalgal treatments resulted in a higher dry mass investment into leaves when compare with the two controls. On the contrary, 70% and 20% LA showed greater investment of dry mass in the stem, with investment of 44.2 and 46.4% of dry mass in this organ, respectively, while the treatments with microalgae did not exceeded 43%. The investment in inflorescence in 70% LA was the highest with 9.2%, in the other treatments was between 5.6% and 6.6%. In Figure 2B, it can be seen that the increase in the amount of wet microalgae applied resulted in an increase in the amount of dry mass of shoot, being greater than the mass obtained by 20% LA. However, the observed increase from the 3 g to 6 g of microalgae was not significantly different. The application of 9 g of wet microalgae stands out among them, presenting 40% more dry mass when compared to 20% LA, and an increase in the dry mass of stem, leaves and inflorescence can be observed.

Application of 3 g of biosolid did not result in a significant increase in shoot dry mass compared to 20% LA (Fig. 2C). However, application of 6 and 10 g plus 20% LA resulted in a significant increase of 28% and 47%, respectively, when compared to 20% LA. It is important to note that the biosolid alone was able to produce about 54% of the dry mass of shoot of the 20% LA control, and about 18% of the 70% LA control. In relation to dry matter partitioning in the above ground, the addition of increasing doses of biosolid resulted in less investment of dry mass into stem from 45% for 3 g of biosolid plus 20% LA to 33% under pure biosolid when compared to 20% control (Fig. 2C). Contrary results were found for the investment in dry mater in the leaves. The investment in dry matter in leaves was from 49% to 61% when compare with 20% LA. However, the addition of biosolid did not result in significant alteration in the proportion of the dry matter investment in inflorescence.

At the end o experiment, the plants grown under only reclaimed water produced 26.5% of the dry mass of the aerial part produced by the control 20% LA, and of the total mass produced about 64% is represented by the leaves, 33.5% by the stem and 2.6% by the inflorescence (Fig. 2D). In the 20% LA control, the investment in dry mass in stem and leaves was similar (48% and 46%, respectively). In relation to the 70% LA control, the plants supplied only with reclaimed water produced about 9% of the dry matter of the above ground.

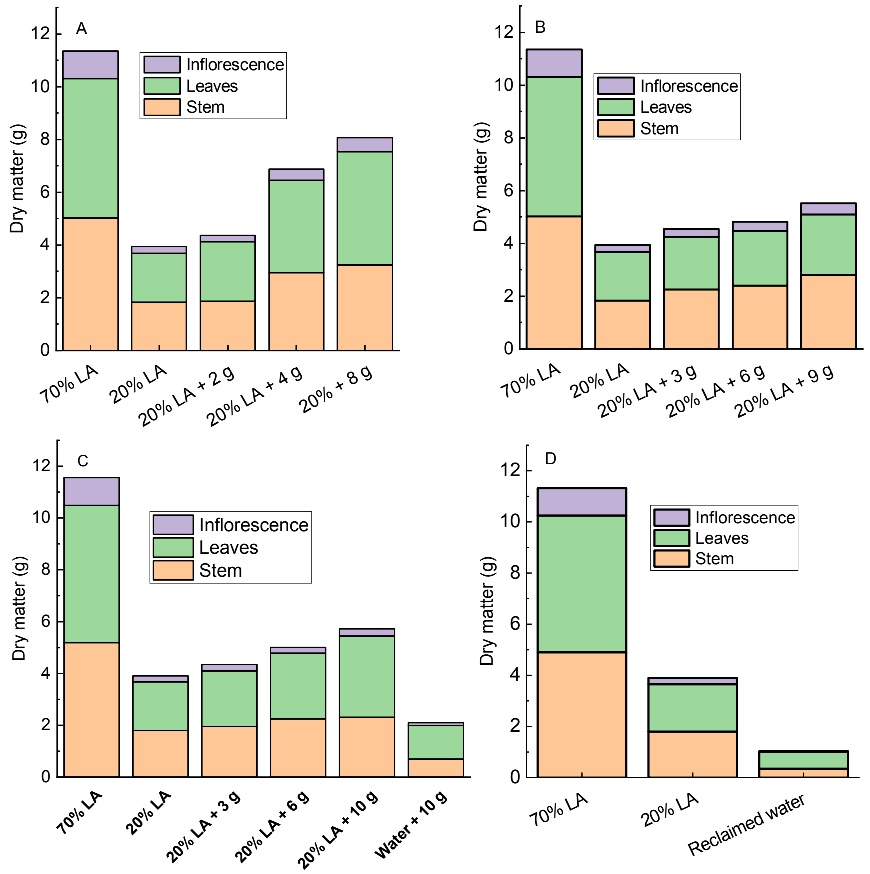

Fig 2. Dry matter partitioning in the above ground of amaranth plants grown under addition of dry microalgae (A), wet microalgae (B), biosolid (C) or reclaimed water (D) after 56 days of sowing. Values are mean of 8 plants.

Photosynthetic pigments and Gas exchange characteristics

Plants grown under 20% LA showed a reduction of about 30% in total chlorophyll and total carotenoids compared to 70% LA (Table 1). The content of total chlorophyll was significantly lower in plants supplied with 4 g of dry microalae when compared with 20% LA and with the application of 8 g of dry microalgae. The total carotenoids was significantly reduced under 20% LA without or with the addition of microalgae when compared to 70% LA. However, the addition of 8 g of microalgae resulted in an increase in total carotenoids compared to 20% LA without microalgae, with no alteration in the other treatments. On the other hand, addition of wet microalgae was not able to maintain the same content of photosynthetic pigments as the 20% LA without wet microalgae.

Table 1. Total chlorophyll and carotenoids of amaranth leaves. Dm, dry microalgae; Wm, wet microalgae. Mean values sharing the same letter within each treatment group do not differ significantly at P = 0.05.

Treatments	Chl[illegible]	Carotenoids
70% LA + 0 g	0.270[illegible]	0.081±0.003 a
20% LA + 0 g	0.190[illegible]	0.058±0.002 c
20% LA + 2 g Dm	0.179±0.016 bc	0.057±0.003 c
20% LA + 4 g Dm	0.157±0.013 c	0.053±0.000 c
20% LA + 8 g Dm	0.218±0.008 ab	0.069±0.002 b
70% LA + 0 g	0.270±0.016 a	0.081±0.003 a
20% LA + 0 g	0.190±0.007 b	0.058±0.002 b
20% LA + 3 g Wm	0.143±0.005 c	0.045±0.001 c
20% LA + 6 g Wm	0.145±0.005 c	0.046±0.001 c
20% LA + 9 g Wm	0,141±0.008 c	0.046±0.002 c
70% LA + 0 g	0.270±0.016 a	0.081±0.003 a
20% LA + 0 g	0.190±0.007 c	0.058±0.002 bc
20% LA + 3 g biosolid	0.153±0.012 c	0.045±0.003 d
20% LA + 6 g biosolid	0.188±0.024 cd	0.054±0.006 cd
20% LA + 10 g biosolid	0.236±0.012 ab	0.067±0.003 b
Water + 10 g biosolid	0.218±0.006 bd	0.066±0.001 b
70% LA + 0 g	0.270±0.016 a	0.081±0.003 a
20% LA + 0 g	0.190±0.007 b	0.058±0.002 b
Reclaimed water	0.240±0.014 a	0.067±0.004 b

The table should be self explanatory, the units of parameters are missing

Addition of 3 g and 6 g of biosolid did not result in a significant change in total chlorophyll content when compared with the 20% LA control (Table 1). Otherwise, application of 10 g plus 20% LA and 10 g of pure biosolid resulted in a significant increase of 22% and 13%, respectively, in total chlorophyll content when compared to the 20% LA control. The total chlorophyll content in the treatment with 10 g of biosolid plus 20% LA reached a value similar to the control 70% LA. Application of 3 g of biosolid resulted in lower carotenoids content compared to 20% LA control, 10 g biosolid plus 20% LA and 10 g of biosolid plus tap water. However, the other biosolid treatments did not differ from the 20% LA control.

Plants supplied only with reclaimed water showed a significant increase of 24% in the total chlorophyll content in relation to the 20% LA (Table 1). Comparing the plants under reclaimed water with the plants under 70% LA, application of reclaimed water resulted in about 87% of the total chlorophyll content found in the control 70% LA. The total carotenoids of plants grown under reclaimed water were similar to 20% LA.

The rate of photosynthesis (A) was significantly reduced under 20% LA compared to 70% LA (Fig. 3A). Addition of 2 g of dry microalgae induced an increase in A above the values found in plants under 20% LA, becoming equal to the 70% LA. Comparing A of the plants grown under addition of dry microalgae with the 20% LA, only the addition of 2 g and 8 g of dry microalgae showed an increase in A. Plants grown under 20% LA resulted in a significant increase in stomatal conductance (g_s) compared with 70% LA (Fig 3B). However, addition of 2 g and 4 g showed lower g_s than the control 20% LA while under 8 g of dry microalgae the g_s was higher than in 20% LA. The concentration of CO_2 in the substomatal cavity (C_i) was significantly lower in plants grown under 70% LA compare to 20% LA (Fig. 3C). The addition of 4 g and 8 g of dry microalgae did not alter the C_i compared to 20% LA. However, addition of 2 g of dry microalgae resulted in lower C_i. The addition of 2 g of microalgae resulted in values of C_i similar to 70% LA. The treatments with wet microalgae showed a lower A than the controls 20% LA, with 3 g having the lowest rate (Fig. 3D). The rate of g_s also presented values below the two controls for the treatments with wet microalgae (Fig. 3E). Despite the g_s being smaller in plants grown under addition of wet microalgae than the in the 20% LA, the C_i it was higher or equal to the control 20% LA (Fig. 3F).

Application of 3 g and 6 g of biosolid resulted in no change in A when compared to 20% LA control (Fig. 3G). However, application of 10 g with or without 20% LA resulted in an increase in A, surpassing the A values of the 70% LA control. Therefore, the addition of 10 g of biosolid promoted A when compared to the two controls. Biosolid application with or without 20% LA resulted in a significant decrease in g_s when compared to 20% LA control (Fig. 3H). Among the biosolid doses there was a positive correlation between biosolid dose and g_s. Application of 10 g of biosolid with tap water resulted in g_s values similar to the 70% LA control, while the g_s in the 20% LA control was higher than in all other treatments. The application biosolid at the different doses resulted in a reduction in C_i when compared to 20% LA and 70% LA controls (Fig. 3I). It is important to note that although the g_s increased with the increase in the biosolid doses, the C_i was reduced.

Although the reclaimed water does not have an ideal balance of nutrients, the value of A was 17.21 μmol m^{-2} s^{-1}, a reduction of 20% and of 33% when compared to 20% and 70% LA, respectively (Fig. 3J). Plants grown under reclaimed water presented a significant reduction in g_s of 37% compared to 20% LA (Fig. 3K). However, when compared to 70% LA the reduction was only 20%. The C_i for the plants grown under reclaimed water did not differ significantly from the 20% control (Fig. 3L).

Chemical composition of biofertilizers and leaf of amaranth

The characterization of the dry microalgae used as a fertilizer in this experiment shows that its nutrient content is unbalanced in relation to plant needs (Table 2). The biomass of these microalgae has an adequate content of N, Ca, P and S, while K, Mg and Mn had a concentration below the requirements of a proper amaranth growth. In terms of micronutrients, the imbalance is shown by the content of Na, Fe, Zn and Cu that are above the appropriate level. The analysis of wet microalgae shows lower levels of macronutrients compared the content of macronutrients found in the leaves of well nourish plants (Table 2). In relation to micronutrients content, Mn presented a lower level while Cu, Fe and Zn were found in higher concentration. The content of Na was within ideal levels for amaranth.

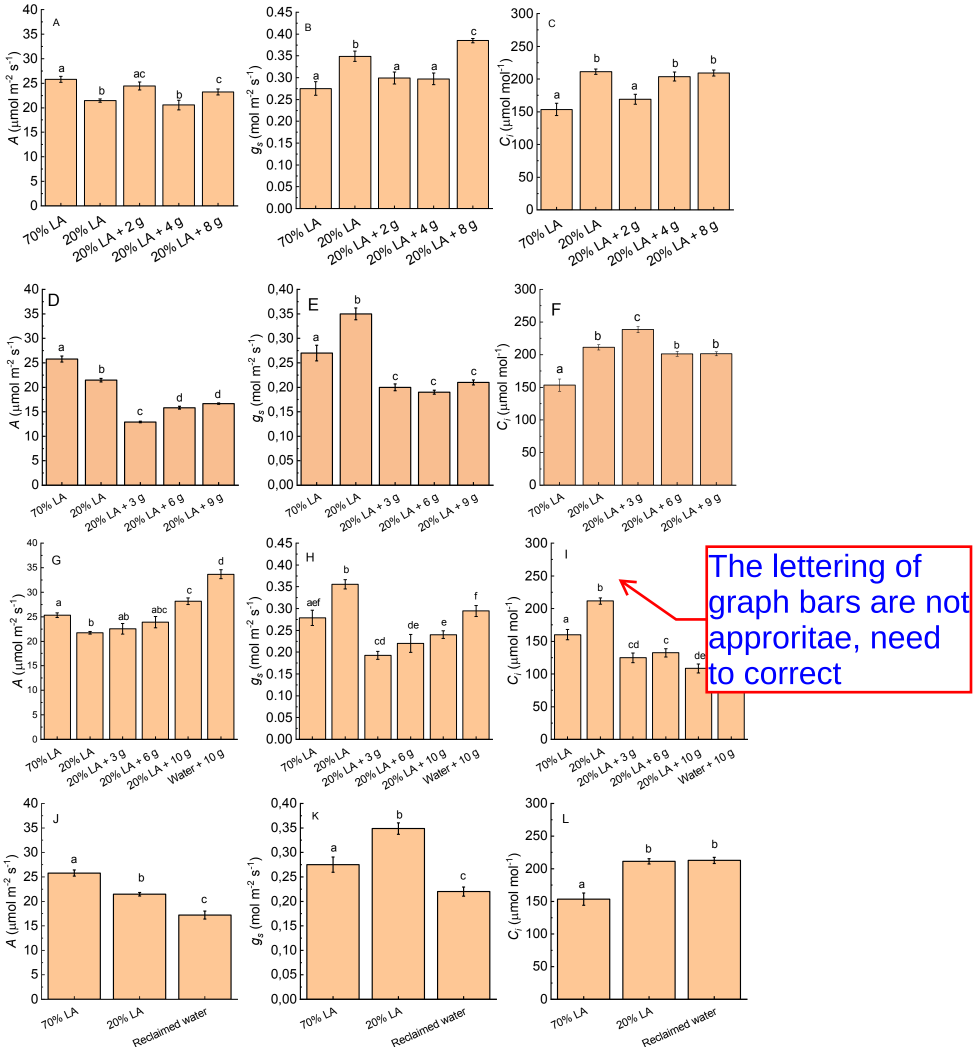

Fig 3. Photosynthesis (A; A, D, G, J), stomatal conductance (g_s; B, E, H, K), and intercellular CO_2 concentration (C_i, C,F, I, L) of amaranth plants grown under addition of dry microalgae (A, B, C), wet microalgae (D, E, F), biosolid (G, H, I) or reclaimed water (J, K, L) after 43 days of sowing. Values are mean±SE of 8 plants.

The chemical analysis of the biosolid reveal that the macronutrients content of N, K, Mg and S are below the suitable levels when compared with the plants grown under optimal availability of nutrients (70% LA), while the content of P and Ca are above the suitable level, respectively (Table 2). The content of micronutrients Cu, Fe, Zn and Na are above the level found in the plants grown under suitable nutrient supply (70% LA). The content of Co, Cr, Ni, Pb and Se were below the tolerable limit (data not shown). On the contrary, the content of Mn was lower. In the Table 2 the chemical analysis of reclaimed water shows that it contains significant amounts of N, K, Ca and S after the wastewater treatment with microalgae but lower amounts of P and Mg. In relation to micronutrients, these are found in significantly lower concentrations.

As control treatments, we used plants grown under supply of 70% or 20% full strength LA nutrient solution in the absence of microalgae (70% LA and 20% LA). In previous experiments it was observed that desirable plant growth was achieved with the application of the 70% nutrient solution [42]. Leaves are plant organs that best reflect

Table 2. Nutrient content of biofertilizers from wastewater treatment and leaves of amaranth plants. Dm, dry microalgae; Wm, wet microalgae; BDL, below detection level.

**Variables**	**Macronutrients (g Kg^{-1})**						**Micronutrients (mg Kg^{-1})**					
	N	P	K	Ca	Mg	S	B	Cu	Fe	Mn	Zn	Na
Biofertilizers												
Dry microalgae (Dm)	57	14.2	5.2	6.8	3.1	4.0	-	202	5572	102	2925	20803
Wet microalgae (Wm)	5.6	1.3	0.1	0.4	0.2	0.4	-	27.7	510	13	291	164
Biosolid	24.4	13.3	0.9	8.4	2.2	2.8	-	177	24752	115	3167	858
Reclaimed water (mg L^{-1})	49.0	2.0	151	13	2.0	9.4	0.19	BDL	0.08	BDL	0.01	47.7
Amaranth leaves												
70% LA + 0 g	28	6.4	60	7	13.9	3.9	24	15	229	359	36	170
20% LA + 0 g	20	4.6	39	7	18.7	4.9	35	9	115	252	25	160
20% LA + 2 g Dm	23	3.8	48	5	20.0	6.0	27	10	93	401	37	120
20% LA + 4 g Dm	25	5.2	39	7	19.8	3.9	36	9	82	98	39	140
20% LA + 8 g Dm	29	6.4	42	6	21.1	4.0	39	11	89	103	43	190
20% LA + 3 g Wm	22	5.1	35	11	24.1	5.4	42	10	86	152	27	180
20% LA + 6 g Wm	22	5.0	36	12	24.9	4.8	45	9.0	78	176	30	180
20% LA + 9 g Wm	20	4.6	32	11	23	5.0	45	11	75	186	43	170
20% LA + 3 g biosolid	21	4.9	37	9	20.2	4.3	42	13	84	239	105	140
20% LA + 6 g biosolid	22	4.7	45	8	18.1	3.7	38	14	101	252	126	130
20% LA + 10 g biosolid	25	4.9	46	7	16.0	3.0	41	17	138	221	198	140
Water + 10 g biosolid	20	3.4	32	7	16.2	6.8	28	17	142	224	222	120
Reclaimed water	26	6.3	44	9	26.2	3.7	25	3	90	37	2	-

- not determined

the nutritional status of the plant. Therefore, they indicate changes in the availability of nutrients in the soil. The chemical analyses of nutrients in the leaves were done 56 days after sowing. Plants grown under 20% LA showed lower levels of almost all nutrients analysed compare to 70% LA (Table 2). The leaf chemical analysis show that of all the doses of dry microalgae used, the best results were found in the dose of 8 g, which showed superior results than the 20% LA for most macronutrients, except for S. The contents of micronutrients B, Cu, Zn and Na were increased under the application of 8 g of dry microalgae compared to 20% LA. It is interesting to note that even the content of Cu, Fe, Zn and Na were higher in the dry microalgae, no excess of this nutrients were found in the leaves of amaranth. There was no clear correlation between the dose of wet microalgae applied and the concentration of nutrients observed in the leaves. Application of 9 g of wet microalgae resulted in higher levels of Ca, Mg, B, Cu, Zn and Na in the leaves of amaranth when compared to 20% LA (Table 2). When compared to the 70% control, the values for Ca, Mg, B and Zn were higher in plants with 9 g of wet microalgae. On the other hand, the content of K, Fe and Mn were lower than the two controls.

The content of N, K, B, Cu and Zn in the leaves of amaranth under the addition of 6 and 10 g of biosolid plus 20% LA were increase compare to 20% LA while P and Ca did not change significantly (Table 2). The content of Mg and S were decreased with the increase in the doses of biosolid compared with 20% LA. Although the content of Na was high in the sludge its content was reduced to below the content of 20% LA. The Mn content under 10 g of biosolid with or without LA were reduced. When 10 g of biosolid were applied with tap water, the content o P, K, Mg, B and Mn were decreased while the content of S was increased. The high content of Cu, Fe and Zn in the biosolid resulted in more concentration in the leaves compared with the control 20% LA. Although the supply of Na in the biosolid was high, this did not result in a further increase in the concentration in the amaranth leaves. The leaves under reclaimed water presented contents of N, P, K, Ca and Mg that were higher than in the 20% LA (Table 2). However, the levels of micronutrients were much lower. Comparing reuse water with 70% LA, the leaves from plants grown under reclaimed water had lower concentrations of N and K but higher concentrations of Ca and Mg.

Discussion

Various methods to replace chemical fertilization have been explored, including the use of secondary products from different stages of sanitary sewage treatment that provide a rich source of nutrients. This study focuses on the use of sludge generated from anaerobic treatment of sanitary sewage, microalgae biomass as post-treatment of sanitary sewage, and reclaimed water obtained after separating microalgae as alternatives for recovering and using nutrients as fertilizers in amaranth cultivation.

The generation and appropriate disposal of sludge produced during the treatment of sanitary sewage has been a topic of concern, with efforts to reduce its production [11] and to find sustainable reuse options. In Brazil, for instance, the annual production of dry matter from sludge exceeds 150,000 tons [43]. Fertilization has been identified as a promising destinations found for the sludge, with laws and regulations in place to ensure safety parameters for its application as biosolids [44, 45]. The sludge and microalgae are rich sources of nutrients, particularly nitrogen, phosphorus, and micronutrients [26, 46, 47]. Moreover, the application of microalgae in soil can improve soil organic matter, which can enhance plant productivity [6, 48, 49]. Combining microalgae cultivation with sanitary sewage treatment can improve the cost-benefit of both processes [50]. The treated effluent obtained after the cultivation of microalgae is a crucial aspect of sanitary sewage treatment, and its use in agricultural practices can

reduce the use of potable water for irrigation and help to recycle remaining nutrients that would otherwise be released into water bodies.

The nutrient composition of the organic fertilizers used in this study revealed that they all contained nutrients essential for plant development, albeit at different concentrations depending on the stage of treatment. The anaerobic sludge has lower levels of macronutrients such as N and K, compared to the dry microalgae biomass. However, the N and Ca content of the biosolid were suitable for the requirements of amaranth. The metal analysis showed that due to its high Zn concentration, the biosolid used was classified as class 2 by CONAMA resolution Nº 498/2020, which restricts the maximum annual application rate based on the soil's characteristics [45]. The other metals analyzed are below the established thresholds. Similar to studies by [51] and [52], the accumulation of Zn in the leaves of plants that received biosolids occurred together with copper accumulation. Zinc toxicity can reduce the leaf nutrient content, particularly for K, Fe and Mg, by inhibiting assimilation and interfering with nutrient translocation to the leaves [53–55]. The high Zn concentration in the biosolid might have affected the assimilation and/or translocation of Fe, which was present in high concentrations in the biosolid but did not accumulate in the leaf tissues, it might have also influenced the leaf Mn content that did not exceed 20% the content in the treatment.

The nutrient composition of microalgae biomass depends on various cultivation conditions, such as temperature, luminosity, and sewage characteristics. These factors influence the composition of microalgae community, and different species of microalgae have different capacities for nutrient removal [56–58]. Additionally, the native species of sanitary sewage are well-adapted to that environment, and changes in the community can occur based on different seasons [59, 60]. While microalgae have shown to have nutrients available for the development of amaranth, wet microalgae contains smaller amounts of these nutrients. Despite the high concentration of micronutrients such as Cu, Fe, Zn and Na in the dry microalgae biomass and in the sludge, there was no significant increase in the leaves. This may indicate that the plant has specific mechanisms to limit the absorption of excess nutrients and avoid physiological damage [61] or the presence of interactions between different nutrients [62, 63]. For example, in the plants that received the biosolid, the high concentration of Zn in the microalgae biomass may have interfered with the assimilation and translocation of nutrients such as Mn and Fe, leading to leaf concentrations lower than the 20% control. It is possible that the foliar concentration of Zn was not high in these treatments, but the inhibition could have occurred in the roots, as demonstrated by [64] where Zn and Cu showed a marked accumulation in the roots in relation to the shoot, which represents a plant strategy to mitigate toxicity by these metals.

Saline stress conditions can induce nutrient deficiencies in leaves, such Fe and Mn [65]. Additionally, this condition is often accompanied by an increase in foliar Na concentration and a decrease in K and N [66]. However, in the present study, with 8 g of dry microalgae and the treatments with wet microalgae showed an elevated concentration of Na in relation to the control treatments.

There were no other signs of saline stress conditions, indicating that the nutrient deficiencies found in this study may not be related to salt stress conditions. It is noteworthy that Na is considered an essential nutrient in C_4 plants and is involved in various physiological processes, such as the recovery of phosphoenolpyruvate and the synthesis of chlorophyll [67–69]. For a more precise investigation in relation to the stress caused by nutrition, proline analysis could be a good indicator, as it has been observed that an increase in proline production is related to stress situations [70, 71]. Another possibility is that the micronutrients may not have been fully released. According to [72], microalgae can be used as a slow-release organic fertilizer, releasing nutrients gradually.

However, the mechanism of nutrient release by microalgae requires further investigation.

Reclaimed water, in turn, due to the efficiency of nutrient removal by microalgae, has a lower nutrient content compared to the other organic fertilizers in this study. The macronutrient content, mainly N and K, is significant and present in higher concentrations than in the 20% nutrient solution, although nutrient values may vary considerably for reuse water depending on environmental characteristics and the treatment the effluent receives. The values found in this study were similar to those obtained by [73], [74], who used conventional treatments, and [75] whose treatment also occurred with the use of microalgae.

One of the major concerns regarding the application of fertilizers from the treatment of sanitary sewage is the potential presence of contaminants such as pathogens and heavy metals. Different studies demonstrate the efficiency of microalgae in disinfecting the environment by varying the conditions such as pH and oxygen concentration, and by intra-algal competition for nutrients and light [24, 76, 77]. Moreover, exposure to the sunlight and the drying process at temperatures above 55^0C inactivate bacteria such as *Escherichia coli* and disinfect the effluent [78–80], thus ensuring the safety of products derived from sewage treatment for use in agriculture. The sludge generated from domestic sewage tends to have concentrations below the safety standards established by CONAMA [81, 82].

Plant growth is closely related to the availability of nutrients. The amaranth genus is known for its fast growth, with an accelerated growth phase occurring 30 days after emergence, and reaching physiological maturity 90 days after emergence [37]. In general, plants that received organic fertilizers together with 20% LA solution showed greater growth throughout the experiment, resulting in higher dry mass production. In addition to any mechanism of interaction between the different nutrients or of existing absorption, the accelerated growth may also indicate nutrient depletion from the organic fertilizers, which may not have been sufficient to meet the plant's nutrient requirements during the entire growth cycle, leading to nutrient deficiencies. Although the mineral content of the reclaimed water was lower than that of the 20% LA control solution, the plants that received only the former produced approximately 27% of the dry mass generated by the latter. This suggests that reclaimed water has potential as a biofertilizer Similarly, plants that received only sludge showed the potential to produce dry mass with the nutrients available in the sludge. Our study aligns with other studies that demonstrated the efficiency of using microalgae, biosolid and reclaimed water as biofertilizers for the growth of various crops [27, 51, 83, 84].

The low production of dry mass under pure sludge treatment may attributed to the low supply of K by the sludge, as the plants did not receive 20% LA. K plays an essential role in maintaining water status and the translocation and transformation of sugars, which directly impacts plant growth and development [85, 86]. On the other hand, the low production of dry mass in the treatment that exclusively received the reclaimed water, is mainly due to the deficiency of micronutrients, since Cu, Fe, Mn and Zn are below the concentrations of the 20% LA control, while the other nutrients reached the concentrations of the control treatment. As micronutrients play a crucial role in plant growth, development and metabolism, the deficiency of these essential nutrients negatively impacts dry mass production [87].

High levels of chlorophyll are essential for normal photosynthesis in plants. The adequate supply of nutrients, particularly N and Mg, is necessary for the synthesis of chlorophyll, as they are part of the molecule. Plants grown under wet microalgae showed a significant reduction in chlorophyll content even with adequate foliar N and Mg content, indicating that a large portion of these nutrients did not contribute to chlorophyll synthesis. When compared to plants grown under dry microalgae, those grown under wet microalgae had much lower foliar Fe content. Chloroplasts have the

highest requirement of Fe in plant cells, comprising up to 80% of the cellular Fe in leaf cells [88]. Fe is essential for the biosynthesis of chlorophyll and its precursors and for the maintenance of the structure and function of chloroplasts [89–91]. Hence, the lower concentrations of Fe in the plants grown under wet microalgae could have been responsible for the reduction in chlorophyll content. The lower photosynthetic pigments in this group of plants may be a consequence of rapid initial growth of plants, leading to rapid exhaustion of available nutrients in the wet microalgae with time. Although excessive Cu and Zn concentrations can significantly reduce chlorophyll content in plants [90, 92–95], this was not observed in plants grown under dry microalgae or sludge, which contained higher concentrations of Cu and Zn. The sludge had four times more Fe than in the dry microalgae in its composition, resulting in a higher content of Fe and chlorophyll in the leaves, highlighting the role of Fe in the chlorophyll biosynthesis. Photosynthetic pigments were consistently higher in crops watered with the treated wastewater [84]. In our study, plants cultivated with reclaimed water, chlorophyll content was higher than than those watered with 20% LA, possibly due to the increase in N and Mg levels, which are part of the chlorophyll molecule. Additionally, the observed decrease of carotenoids content in the leaves of plants cultivated under wet microalgae can be attributed to the role of Fe in carotenoid biosynthesis [90].

Photosynthesis can be limited by stomatal and non-stomatal factors. If CO_2 conductance is the only factor, limiting photosynthesis, C_i would be expected to decrease due to CO_2 removal from intercellular spaces and decreased resupply through stomata. However, this was not observed in the plants grown under wet microalgae, where C_i was increased or maintained at the same values as 20% LA control. This suggests that the reduction in photosynthesis was not solely due to stomatal closure but also due to lower levels of photosynthetic pigments and other non-stomatal limitations. Interestingly, despite the lower photosynthetic rate observed after 43 days of sowing, plants grown under wet microalgae showed an increase in shoot dry matter at the end of the experimental period. The effects of metals on photosynthetic capacity depends on their concentration availability. Lower concentrations of Zn have been shown to increase photosynthetic rate, g_s, E and chlorophyll levels, while higher concentrations decreased them [96, 97]. Copper is an essential micronutrient for various metabolic processes in plants, but high concentrations can have a toxic effect on photosynthetic and biochemical parameters [98]. In our study, however, high doses of Cu and Zn from dry microalgae or sludge did not decrease photosynthesis. On the contrary, photosynthesis increased as the doses of dry microalgae and sludge increased. Our findings are consistent with previous reports that application of sewage sludge can increase photosynthesis, chlorophyll content, and Fe acquisition [44]. While plants grown under sludge supply showed lower g_s, photosynthesis increased, resulting in lower C_i, indicating effective CO_2 assimilation in the substomatal cavity. Plants grown solely with reclaimed water showed a 80% photosynthetic rate compared to the 20% LA control. However, the g_s was lower, and C_i reached a value similar to the 20% LA control, suggesting that the non-stomatal limitation was due to micronutrients deficiency in the reclaimed water.

Conclusion

In conclusion, our study highlights the potential of organic fertilizers from domestic sewage treatment, such as biosolids, wet and dry microalgae, to enhance the biomass of aerial parts of amaranth. Despite the nutrient deficiencies observed in some treatments, the application of larger doses of these products significantly increased biomass production, with dry microalgae being the most effective. While the treatments receiving exclusively reuse water or sludge did not surpass the control treatments, they

demonstrated the potential for biomass production with available nutrients. However, to optimize initial growth, further investigations are needed to determine the appropriate doses and application rates of these products. Additionally, the high concentrations of micronutrients such as Cu, Fe, Zn, and Na in the biosolids and microalgae biomass require careful consideration to avoid potential stress on plants. Overall, our findings suggest that organic fertilizers from domestic sewage treatment can offer a sustainable solution for nutrient cycling by returning them to the soil and promoting plant growth in nutrient-poor soils.

Acknowledgements

Author GHRS thanks the Brazilian agency FAPESP for financial support of the main project (Grant number: 18/18367-1), and the authors ETF and SCB thank for the undergraduate scholarships (Grant numbers: 2020/06459-9, 2020/10764-1, respectively). The author GHRS also thanks the Brazilian agency CNPq for financial support of the main project (Grant numbers: 308663/2021-7, 309064/2018-0, 427936/2018-7) and SPM thanks for the undergraduate scholarship (Grant number: 334). The authors thank the Department of Water and Sewage/Bauru for allowing us to carry out the research. The authors thank TANAC for providing the tannin.

References

1. Sariatli F. Linear economy versus circular economy: a comparative and analyzer study for optimization of economy for sustainability. Visegrad Journal on Bioeconomy and Sustainable Development. 2017;6(1):31–34.

2. Babbitt CW, Gaustad G, Fisher A, Chen WQ, Liu G. Closing the loop on circular economy research: From theory to practice and back again. Resources, Conservation and Recycling. 2018;135:1–2.

3. Morseletto P. Targets for a circular economy. Resources, Conservation and Recycling. 2020;153:104553.

4. Hussain F, Shah SZ, Ahmad H, Abubshait SA, Abubshait HA, Laref A, et al. Microalgae an ecofriendly and sustainable wastewater treatment option: Biomass application in biofuel and bio-fertilizer production. A review. Renewable and Sustainable Energy Reviews. 2021;137:110603.

5. Silambarasan S, Logeswari P, Sivaramakrishnan R, Incharoensakdi A, Cornejo P, Kamaraj B, et al. Removal of nutrients from domestic wastewater by microalgae coupled to lipid augmentation for biodiesel production and influence of deoiled algal biomass as biofertilizer for *Solanum lycopersicum* cultivation. Chemosphere. 2021;268:129323.

6. Kopittke PM, Menzies NW, Wang P, McKenna BA, Lombi E. Soil and the intensification of agriculture for global food security. Environment international. 2019;132:105078.

7. Verstraete W, Van de Caveye P, Diamantis V. Maximum use of resources present in domestic "used water". Bioresource technology. 2009;100(23):5537–5545.

8. Mihelcic JR, Fry LM, Shaw R. Global potential of phosphorus recovery from human urine and feces. Chemosphere. 2011;84(6):832–839.

9. Suleiman AKA, Lourenço KS, Clark C, Luz RL, da Silva GHR, Vet LE, et al. From toilet to agriculture: Fertilization with microalgal biomass from wastewater impacts the soil and rhizosphere active microbiomes, greenhouse gas emissions and plant growth. Resources, Conservation and Recycling. 2020;161:104924.

10. Eid EM, El-Bebany AF, Taher MA, Alrumman SA, Galal TM, Shaltout KH, et al. Heavy metal bioaccumulation, growth characteristics, and yield of *Pisum sativum* L. grown in agricultural soil-sewage sludge mixtures. Plants. 2020;9(10):1300.

11. Collivignarelli MC, Canato M, Abba A, Miino MC. Biosolids: what are the different types of reuse? Journal of Cleaner Production. 2019;238:117844.

12. Ashekuzzaman S, Forrestal P, Richards K, Fenton O. Dairy industry derived wastewater treatment sludge: Generation, type and characterization of nutrients and metals for agricultural reuse. Journal of Cleaner Production. 2019;230:1266–1275.

13. Rekik I, Chaabane Z, Missaoui A, Bouket AC, Luptakova L, Elleuch A, et al. Effects of untreated and treated wastewater at the morphological, physiological and biochemical levels on seed germination and development of sorghum (*Sorghum bicolor* L Moench), alfalfa (*Medicago sativa* L.) and fescue (*Festuca arundinacea* Schreb.). Journal of Hazardous Materials. 2017;326:165–176.

14. Eid E, El-Bebany A, Taher M, Alrumman S, Hussain A, Alghamdi A, et al. EFFECTS OF SEWAGE SLUDGE APPLICATIONS TO AGRICULTURAL SOIL ON THE BIOCHEMICAL PARAMETERS OF FABA BEAN (*Faba sativa* Bernh.), WHEAT (*Triticum aestivum* L.), SPINACH (*Spinacia oleracea* L.) AND CUCUMBER (*Cucumis sativus* L.) CROPS. Applied Ecology and Environmental Research. 2020;18(5):6457–6467.

15. Gubišová M, Horník M, Hrčková K, Gubiš J, Jakubcová A, Hudcovicová M, et al. Sewage Sludge as a Soil Amendment for Growing Biomass Plant *Arundo donax* L. Agronomy. 2020;10(5):678.

16. Halecki W, López-Hernández NA, Koźmińska A, Ciarkowska K, Klatka S. A Circular Economy Approach to Restoring Soil Substrate Ameliorated by Sewage Sludge with Amendments. International Journal of Environmental Research and Public Health. 2022;19(9):5296.

17. Poornima R, Suganya K, Sebastian SP. Biosolids towards Back–To–Earth alternative concept (BEA) for environmental sustainability: a review. Environmental Science and Pollution Research. 2022; p. 1–42.

18. Zeeman G, Kujawa K, De Mes T, Hernandez L, De Graaff M, Abu-Ghunmi L, et al. Anaerobic treatment as a core technology for energy, nutrients and water recovery from source-separated domestic waste (water). Water Science and Technology. 2008;57(8):1207–1212.

19. Li K, Liu Q, Fang F, Luo R, Lu Q, Zhou W, et al. Microalgae-based wastewater treatment for nutrients recovery: A review. Bioresource technology. 2019;291:121934.

20. Slompo NDM, Quartaroli L, Zeeman G, da Silva GHR, Daniel LA. Black water treatment by an upflow anaerobic sludge blanket (UASB) reactor: a pilot study. Water Science and Technology. 2019;80(8):1505–1511.

21. Acién Fernández FG, Gómez-Serrano C, Fernández-Sevilla JM. Recovery of nutrients from wastewaters using microalgae. Frontiers in Sustainable Food Systems. 2018;2:59.

22. Moges ME, Heistad A, Heidorn T. Nutrient recovery from anaerobically treated blackwater and improving its effluent quality through microalgae biomass production. Water. 2020;12(2):592.

23. de Wilt A, Butkovskyi A, Tuantet K, Leal LH, Fernandes TV, Langenhoff A, et al. Micropollutant removal in an algal treatment system fed with source separated wastewater streams. Journal of hazardous materials. 2016;304:84–92.

24. Ruas G, Serejo M, Farias S, Scarcelli P, Boncz M. Removal of pathogens from domestic wastewater by microalgal-bacterial systems under different cultivation conditions. International Journal of Environmental Science and Technology. 2021; p. 1–12.

25. Li Y, Tarpeh WA, Nelson KL, Strathmann TJ. Quantitative evaluation of an integrated system for valorization of wastewater algae as bio-oil, fuel gas, and fertilizer products. Environmental science & technology. 2018;52(21):12717–12727.

26. Renuka N, Prasanna R, Sood A, Bansal R, Bidyarani N, Singh R, et al. Wastewater grown microalgal biomass as inoculants for improving micronutrient availability in wheat. Rhizosphere. 2017;3:150–159.

27. Das P, Quadir M, Thaher M, Alghasal G, Aljabri H. Microalgal nutrients recycling from the primary effluent of municipal wastewater and use of the produced biomass as bio-fertilizer. International journal of environmental science and technology. 2019;16(7):3355–3364.

28. Agrafioti E, Diamadopoulos E. A strategic plan for reuse of treated municipal wastewater for crop irrigation on the Island of Crete. Agricultural water management. 2012;105:57–64.

29. Acosta-Motos JR, Ortuño MF, Álvarez S, Hernández JA, Sánchez-Blanco MJ. The use of reclaimed water is a viable and safe strategy for the irrigation of myrtle plants in a scenario of climate change. Water Supply. 2019;19(6):1741–1747.

30. Lyu S, Wu L, Wen X, Wang J, Chen W. Effects of reclaimed wastewater irrigation on soil-crop systems in China: A review. Science of The Total Environment. 2022; p. 152531.

31. Menegassi LC, Rossi F, Dominical LD, Tommaso G, Montes CR, Gomide CA, et al. Reuse in the agro-industrial: Irrigation with treated slaughterhouse effluent in grass. Journal of Cleaner Production. 2020;251:119698.

32. Tzortzakis N, Saridakis C, Chrysargyris A. Treated wastewater and fertigation applied for greenhouse tomato cultivation grown in municipal solid waste compost and soil mixtures. Sustainability. 2020;12(10):4287.

33. Khaskhoussy K, Kahlaoui B, Misle E, Hachicha M. Impact of Irrigation with Treated Wastewater on Physical-Chemical Properties of Two Soil Types and Corn Plant (*Zea mays*). Journal of Soil Science and Plant Nutrition. 2022; p. 1–17.

34. Rastogi A, Shukla S. Amaranth: a new millennium crop of nutraceutical values. Critical reviews in food science and nutrition. 2013;53(2):109–125.

35. Alemayehu FR, Bendevis M, Jacobsen SE. The potential for utilizing the seed crop amaranth (*Amaranthus* spp.) in East Africa as an alternative crop to support food security and climate change mitigation. Journal of Agronomy and Crop Science. 2015;201(5):321–329.

36. Teixeira MS, Speranza LG, da Silva IC, Moruzzi RB, Silva GHR. Tannin-based coagulant for harvesting microalgae cultivated in wastewater: Efficiency, floc morphology and products characterization. Science of The Total Environment. 2022;807:150776.

37. Spehar CR, Teixeira DL, Cabezas WARL, Erasmo EAL. Amaranth BRS-Alegria: alternative for diversification of cropping systems. Pesquisa agropecuária brasileira. 2003;38:659–663.

38. Hewitt E. Sand and water culture methods used in the study of plant nutrition; 1966.

39. Lichtenthaler HK. [34] Chlorophylls and carotenoids: pigments of photosynthetic biomembranes. In: Methods in enzymology. vol. 148. Elsevier; 1987. p. 350–382.

40. Malavolta E, Vitti GC, Oliveira SAD. Avaliação do estado nutricional das plantas: princípios e aplicações; 1997.

41. Braga AF, Zaiat M, Silva G, Fermoso FG. Metal fractionation in sludge from sewage UASB treatment. Journal of environmental management. 2017;193:98–107.

42. Cechin I, Valquilha ÉM. Nitrogen effect on gas exchange characteristics, dry matter production and nitrate accumulation of Amaranthus cruentus L. Brazilian Journal of Botany. 2019;42(3):373–381.

43. Guimarães MG, Evaristo RBW, Viana NA, Malafaia FR, Itai Y, Brasil AC, et al. Evaluation of Brasilia wastewater sludge as a biomass resource for the production of energy by gasification simulation. International Journal of Sustainable Engineering. 2018;11(1):32–39.

44. Baioui R, Ghnaya T, Wali M, Zaier H, Ghabriche R. Agricultural valorization of domestic sewage sludge: Impact on growth, photosynthesis, nutrition and toxic metal accumulation in Medicago sativa. Agricultural valorization of domestic sewage sludge: impact on growth, photosynthesis, nutrition and toxic metal accumulation in Medicago sativa. 2017; p. 56–74.

45. Brazil. Resolução №498, de 19 de agosto de 2020. Define critérios e procedimentos para produção e aplicação de biossólido em solos, e dá outras providências. Diário Oficial da República Federativa do Brasil. 2020;161:265.

46. Mañas P, De Las Heras J. Nutrient content in wheat grain and straw using sludge and compost from a wastewater treatment plant as a fertiliser. Journal of the Science of Food and Agriculture. 2018;98(12):4707–4714.

47. de Paula Pereira ASA, de Siqueira Castro J, Ribeiro VJ, Calijuri ML. Organomineral fertilizers pastilles from microalgae grown in wastewater: Ammonia volatilization and plant growth. Science of The Total Environment. 2021;779:146205.

48. Renuka N, Guldhe A, Prasanna R, Singh P, Bux F. Microalgae as multi-functional options in modern agriculture: current trends, prospects and challenges. Biotechnology advances. 2018;36(4):1255–1273.

49. Guo S, Wang P, Wang X, Zou M, Liu C, Hao J. Microalgae as biofertilizer in modern agriculture. In: Microalgae biotechnology for food, health and high value products. Springer; 2020. p. 397–411.

50. Delrue F, Álvarez-Díaz PD, Fon-Sing S, Fleury G, Sassi JF. The environmental biorefinery: Using microalgae to remediate wastewater, a win-win paradigm. Energies. 2016;9(3):132.

51. Ceccanti C, Lauria G, Piccolo EL, Guidi L, Pezzarossa B, Rosellini I, et al. Assessment of leaf photosynthetic performances and bioaccumulation of trace metals by lettuce leaves and strawberry fruits amended with sewage sludge: Which possible re-use in agriculture? Scientia Horticulturae. 2022;295:110884.

52. Fei-Baffoe B, Amo-Asare J, Sulemana A, Miezah K. Levels of lead, copper, and zinc in cabbage (*Brassica oleracea* sp.) and lettuce (*Lactuca sativa* sp.) grown on soil amended with sewage sludge. Journal of Environmental and Public Health. 2021;2021.

53. Yilmaz DD, Temizgül A. Effects of municipal sewage sludge doses on the chlorophyll contents and heavy metal concentration of sugar beet (*Beta vulgaris* var. saccharifera). Bioremediation Journal. 2012;16(3):131–140.

54. Garg N, Singh S. Arbuscular mycorrhiza *Rhizophagus irregularis* and silicon modulate growth, proline biosynthesis and yield in *Cajanus cajan* L. Millsp.(pigeonpea) genotypes under cadmium and zinc stress. Journal of plant growth regulation. 2018;37(1):46–63.

55. Kaur H, Garg N. Zinc toxicity in plants: a review. Planta. 2021;253(6):1–28.

56. Caporgno MP, Taleb A, Olkiewicz M, Font J, Pruvost J, Legrand J, et al. Microalgae cultivation in urban wastewater: Nutrient removal and biomass production for biodiesel and methane. Algal Research. 2015;10:232–239.

57. Choudhary P, Prajapati SK, Malik A. Screening native microalgal consortia for biomass production and nutrient removal from rural wastewaters for bioenergy applications. Ecological Engineering. 2016;91:221–230.

58. Chew KW, Chia SR, Show PL, Yap YJ, Ling TC, Chang JS. Effects of water culture medium, cultivation systems and growth modes for microalgae cultivation: A review. Journal of the Taiwan Institute of Chemical Engineers. 2018;91:332–344.

59. Renuka N, Sood A, Prasanna R, Ahluwalia AS. Influence of seasonal variation in water quality on the microalgal diversity of sewage wastewater. South African Journal of Botany. 2014;90:137–145.

60. Nageshwari K, Baishali D, Unpaprom Y, Ramaraj R, Maniam GP, Govindan N, et al. Exploring the dynamics of microalgal diversity in high-rate algal ponds. The Future of Effluent Treatment Plants. 2021; p. 615–660.

61. Aung MS, Masuda H. How does rice defend against excess iron?: Physiological and molecular mechanisms. Frontiers in Plant Science. 2020;11:1102.

62. Maathuis FJ. Sodium in plants: perception, signalling, and regulation of sodium fluxes. Journal of Experimental Botany. 2014;65(3):849–858.

63. Xie K, Cakmak I, Wang S, Zhang F, Guo S. Synergistic and antagonistic interactions between potassium and magnesium in higher plants. The Crop Journal. 2021;9(2):249–256.

64. Tiecher TL, Tiecher T, Ceretta CA, Ferreira PA, Nicoloso FT, Soriani HH, et al. Tolerance and translocation of heavy metals in young grapevine (*Vitis vinifera*) grown in sandy acidic soil with interaction of high doses of copper and zinc. Scientia Horticulturae. 2017;222:203–212.

65. Bosco MRO, de Oliveira AB, Hernandez FF, de Lacerda CF. Influence of salt stress on the mineral composition of eggplant seedlings. Revista Ciência Agronômica. 2009;40(2):157.

66. Wouyou A, Prodjinoto H, Zanklan AS, Vanpee B, Lutts S, Gandonou CB, et al. Implication of ions and organic solutes accumulation in amaranth (*Amaranthus cruentus* L.) salinity resistance. American Journal of Plant Sciences. 2019;10(12):2335.

67. Murata S, Kobayashi M, Matoh T, Sekiya J. Sodium Stimulates Regeneration of Phospho enol pyruvate in Mesophyll Chloroplasts of *Amaranthus tricolor*. Plant and cell physiology. 1992;33(8):1247–1250.

68. Ando T, Oguchi Y, et al. A possible role of sodium in chlorophyll biosynthesis of sodium-requiring C_4-plants. In: Transactions 14th International Congress of Soil Science, Kyoto, Japan, August 1990, Volume IV.; 1990. p. 152–157.

69. Subbarao G, Ito O, Berry W, Wheeler R. Sodium—a functional plant nutrient. Critical Reviews in Plant Sciences. 2003;22(5):391–416.

70. Gill AR, Loveys BR, Cowley JM, Hall T, Cavagnaro TR, Burton RA. Physiological and morphological responses of industrial hemp (*Cannabis sativa* L.) to water deficit. Industrial Crops and Products. 2022;187:115331.

71. Zulfiqar F, Ashraf M. Proline Alleviates Abiotic Stress Induced Oxidative Stress in Plants. Journal of Plant Growth Regulation. 2022; p. 1–23.

72. Coppens J, Grunert O, Van Den Hende S, Vanhoutte I, Boon N, Haesaert G, et al. The use of microalgae as a high-value organic slow-release fertilizer results in tomatoes with increased carotenoid and sugar levels. Journal of applied phycology. 2016;28(4):2367–2377.

73. Alves PFS, dos Santos SR, Kondo MK, Mizobutsi GP, Caldeira LA, Alves IS, et al. Banana fertigation with treated sanitary wastewater: postharvest and microbiological quality. Semina: Ciências Agrárias. 2017;38(3):1229–1240.

74. Santos SR, Ribeiro DP, Matos AT, Kondo MK, Araújo ED. Changes in soil chemical properties promoted by fertigation with treated sanitary wastewater. Engenharia Agrícola. 2017;37:343–352.

75. Urbano VR, Mendonça TG, Bastos RG, Souza CF. Effects of treated wastewater irrigation on soil properties and lettuce yield. Agricultural water management. 2017;181:108–115.

76. Slompo NDM, Quartaroli L, Fernandes TV, da Silva GHR, Daniel LA. Nutrient and pathogen removal from anaerobically treated black water by microalgae. Journal of Environmental Management. 2020;268:110693.

77. Kumar V, Jaiswal KK, Verma M, Vlaskin MS, Nanda M, Chauhan PK, et al. Algae-based sustainable approach for simultaneous removal of micropollutants, and bacteria from urban wastewater and its real-time reuse for aquaculture. Science of The Total Environment. 2021;774:145556.

78. Fogolari O, Reis CZD, Philippi LS. Determinação de parâmetros cinéticos da inativação térmica de *Escherichia coli* em lodo de esgoto. Engenharia Sanitária e Ambiental. 2012;17:255–262.

79. Giannakis S, Darakas E, Escalas-Cañellas A, Pulgarin C. The antagonistic and synergistic effects of temperature during solar disinfection of synthetic secondary effluent. Journal of Photochemistry and Photobiology A: Chemistry. 2014;280:14–26.

80. Gutiérrez-Alfaro S, Rueda-Márquez JJ, Perales JA, Manzano MA. Combining sun-based technologies (microalgae and solar disinfection) for urban wastewater regeneration. Science of the Total Environment. 2018;619:1049–1057.

81. Augustos de Lemos Chernicharo C, Von Sperling M. Biological wastewater treatment in warm climate regions. IWA publishing; 2005.

82. Iloms E, Ololade OO, Ogola HJ, Selvarajan R. Investigating industrial effluent impact on municipal wastewater treatment plant in Vaal, South Africa. International journal of environmental research and public health. 2020;17(3):1096.

83. Wuang SC, Khin MC, Chua PQD, Luo YD. Use of Spirulina biomass produced from treatment of aquaculture wastewater as agricultural fertilizers. Algal research. 2016;15:59–64.

84. Amori PN, Mierzwa JC, Bartelt-Hunt S, Guo B, Saroj DP. Germination and growth of horticultural crops irrigated with reclaimed water after biological treatment and ozonation. Journal of cleaner production. 2022;336:130173.

85. Kanai S, Moghaieb RE, El-Shemy HA, Panigrahi R, Mohapatra PK, Ito J, et al. Potassium deficiency affects water status and photosynthetic rate of the vegetative sink in green house tomato prior to its effects on source activity. Plant science. 2011;180(2):368–374.

86. Thornburg TE, Liu J, Li Q, Xue H, Wang G, Li L, et al. Potassium deficiency significantly affected plant growth and development as well as micro RNA-mediated mechanism in wheat (*Triticum aestivum* L.). Frontiers in Plant Science. 2020;11:1219.

87. Tripathi DK, Singh S, Singh S, Mishra S, Chauhan D, Dubey N. Micronutrients and their diverse role in agricultural crops: advances and future prospective. Acta Physiologiae Plantarum. 2015;37(7):1–14.

88. Yruela I. Transition metals in plant photosynthesis. Metallomics. 2013;5(9):1090–1109.

89. Pushnik JC, Miller GW, Manwaring JH. The role of iron in higher plant chlorophyll biosynthesis, maintenance and chloroplast biogenesis. Journal of Plant Nutrition. 1984;7(1-5):733–758.

90. Lidon FC, Henriques FS. Effects of excess copper on photosynthetic pigments in rice plants. Bot Bull Acad Sinica. 1992;33:141–149.

91. Rout GR, Sahoo S. Role of iron in plant growth and metabolism. Reviews in Agricultural Science. 2015;3:1–24.

92. Li G, Li C, Rengel Z, Liu H, Zhao P. Excess Zn-induced changes in physiological parameters and expression levels of *TaZips* in two wheat genotypes. Environmental and Experimental Botany. 2020;177:104133.

93. Hayat K, Khan A, Bibi F, Murad W, Fu Y, Batiha GES, et al. Effect of Cadmium and Copper Exposure on Growth, Physio-Chemicals and Medicinal Properties of *Cajanus cajan* L. (Pigeon Pea). Metabolites. 2021;11(11):769.

94. Liu J, Deng Q, Shang Y, Yao X, Wang H, Tang Y, et al. Effects of zinc application on the growth and photosynthetic characteristics of pecan at the seedling stage. Plant Biology (Stuttgart, Germany). 2021;.

95. Chen H, Song L, Zhang H, Wang J, Wang Y, Zhang H. Cu and Zn Stress affect the photosynthetic and antioxidative systems of alfalfa (*Medicago sativa*). Journal of Plant Interactions. 2022;17(1):695–704.

96. Fan Y, Jiang T, Chun Z, Wang G, Yang K, Tan X, et al. Zinc affects the physiology and medicinal components of *Dendrobium nobile* Lindl. Plant Physiology and Biochemistry. 2021;162:656–666.

97. Li SP, ZENG LS, SU ZL. Wheat growth, photosynthesis and physiological characteristics under different soil Zn levels. Journal of Integrative Agriculture. 2022;21(7):1927–1940.

98. Mir AR, Alam P, Hayat S. Effect of different levels of soil applied copper on the morpho-physiological, photochemical, and antioxidant system of *Brassica juncea*. Journal of Soil Science and Plant Nutrition. 2021;21(4):3477–3492.
