# Supplementary material for: Biofertilizers from wastewater treatment as a potential source of mineral nutrients for growth of amaranth plants
Source: PLoS One. 2023 Dec 20;18(12):e0295624. doi: 10.1371/journal.pone.0295624 (PMC10732379; doi:10.1371/journal.pone.0295624)
Supplement: Attachment — Submitted filename: Repply to Reviwers.docx [file pone.0295624.s004.docx]

**Dear Editor**

We are pleased that our manuscript has merit to be published in Plos One. We made all the changes suggested by the reviewers. Here goes the reply to your comments.

**Journal Requirements: Brown color in the manuscript.**

1. 1. Please ensure that your manuscript meets PLOS ONE's style requirements, including those for file naming. The PLOS ONE style templates can be found at
   <https://journals.plos.org/plosone/s/file?id=wjVg/PLOSOne_formatting_sample_main_body.pdf> and 
   <https://journals.plos.org/plosone/s/file?id=ba62/PLOSOne_formatting_sample_title_authors_affiliations.pdf>

***Reply:*** *We have used the Plos One LaTeX template obtained from the following web page: https://journals.plos.org/plosone/s/latex.*

1. In your Methods section, please provide additional information regarding the permits you obtained for the work. Please ensure you have included the full name of the authority that approved the field site access and, if no permits were required, a brief statement explaining why.

***Reply:*** *The modification is in the manuscript.*

Modification to the text (topic “Collection of previously treated sewage and and obtaining biosolid”). *The Faculty of Engineering – Bauru at Universidade Estadual Paulista (UNESP) currently maintains a collaborative agreement (code 2100.0632) with the Department of Water and Sewage of Bauru (DAE), which is a foundation of the Municipal Government of the Bauru city. This agreement was officially published in the gazette on May 2, 2023, and is in effect until May 18, 2028. The primary objectives of this agreement refer to the development, dissemination, and practical application of research with the ultimate goal of benefiting society. Under this agreement, researchers are granted access to the sanitary sewage collection site for their research purposes. Furthermore, they are authorized to collect and employ sanitary sewage for their research purposes. Notably, for the cultivation of microalgae and sunflowers, there is no need for legal or institutional permissions, as this activity is an integral component of the researchers’ ongoing research activities.*

3. Thank you for stating the following in the Acknowledgments Section of your manuscript: 
   "Author GHRS thanks the Brazilian agency FAPESP for financial support of the main project (Grant number: 18/18367-1), and the authors ETF and SCB thank for the undergraduate scholarships (Grant numbers: 2020/06459-9, 2020/10764-1, respectively).The author GHRS also thanks the Brazilian agency CNPq for financial support of the main project (Grant numbers: 308663/2021-7, 309064/2018-0, 427936/2018-7) and SPM thanks for the undergraduate scholarship (Grant number: 334). The authors thank the Department of Water and Sewage/Bauru for allowing us to carry out the research. The authors thank TANAC for providing the tannin. "

We note that you have provided funding information that is not currently declared in your Funding Statement. However, funding information should not appear in the Acknowledgments section or other areas of your manuscript. We will only publish funding information present in the Funding Statement section of the online submission form.

Please remove any funding-related text from the manuscript and let us know how you would like to update your Funding Statement. Currently, your Funding Statement reads as follows: 
   "The funders had no role in the study design."

Please include your amended statements within your cover letter; we will change the online submission form on your behalf.

***Reply:*** *There was a misunderstanding in the first submission. We apologise for this. Therefore, please include the following information in the Financial Disclosure section:*

*The project was funded by the* ***São Paulo Research Foundation,*** *FAPESP (Grant numbers: 18/18367-1 [GHRS]; 2020/06459-9 [ETF] and 2020/10764-1 [SCB];* [*https://fapesp.br*](https://fapesp.br) *), the National Council for Scientific and Technological Development (Grant numbers: 308663/2021-7 [GHRS]; 309064/2018-0 [GHRS]; 427936/2018-7 [GHRS] and 334 [SPM];* [*www.cnpq.br*](http://www.cnpq.br)*). The funders had no role in study design, data collection and analysis, decision to publish, or preparation of the manuscript.*

   4. We note that the grant information you provided in the ‘Funding Information’ and ‘Financial Disclosure’ sections do not match.

When you resubmit, please ensure that you provide the correct grant numbers for the awards you received for your study in the ‘Funding Information’ section.

***Reply:*** *Thanks for reminding us of the misunderstanding we mentioned above. In the resubmission process we will fix this ambiguity.*

5. We note that you have indicated that data from this study are available upon request. PLOS only allows data to be available upon request if there are legal or ethical restrictions on sharing data publicly. For more information on unacceptable data access restrictions, please see <http://journals.plos.org/plosone/s/data-availability#loc-unacceptable-data-access-restrictions>.

In your revised cover letter, please address the following prompts:

a) If there are ethical or legal restrictions on sharing a de-identified data set, please explain them in detail (e.g., data contain potentially sensitive information, data are owned by a third-party organization, etc.) and who has imposed them (e.g., an ethics committee). Please also provide contact information for a data access committee, ethics committee, or other institutional body to which data requests may be sent.

b) If there are no restrictions, please upload the minimal anonymized data set necessary to replicate your study findings as either Supporting Information files or to a stable, public repository and provide us with the relevant URLs, DOIs, or accession numbers. For a list of acceptable repositories, please see <http://journals.plos.org/plosone/s/data-availability#loc-recommended-repositories>.

We will update your Data Availability statement on your behalf to reflect the information you provide.

***Reply:*** *All the data related to this study will be available in the following URL address*  <https://doi.org/10.5061/dryad.kprr4xhb5>

6. We note that you have included the phrase “data not shown” in your manuscript. Unfortunately, this does not meet our data sharing requirements. PLOS does not permit references to inaccessible data. We require that authors provide all relevant data within the paper, Supporting Information files, or in an acceptable, public repository. Please add a citation to support this phrase or upload the data that corresponds with these findings to a stable repository (such as Figshare or Dryad) and provide and URLs, DOIs, or accession numbers that may be used to access these data. Or, if the data are not a core part of the research being presented in your study, we ask that you remove the phrase that refers to these data.

***Reply:*** *We removed the phrase that refers to the data of heavy metal content of sludge.*

**Dear Reviewers**

PONE-D-23-17844 - Biofertilizers from wastewater treatment as a potential source of mineral nutrients for growth of amaranth plants

Firstly, we would like to thank the reviewers for their valuable comments on the manuscript. The reviewers’ comments enriched the manuscript making it more readable for the reader. We tried to make the manuscript clearer as much as possible by attending the suggestions made by the reviewers. In what follows, we detail the amendments that we have made in the manuscript as suggested.

**Reviewer #1 –** **Red colour in the manuscript**

1. The research gap has not been highlighted in the need for project section. So it is suggested to add already work done on the similar aspects and what needed

***Reply:*** As suggested by the reviewer, a few references on the subject were added in the manuscript.

**Introduction: beginning of the last paragraph.** Although the use of biofertilizers available after sewage treatment still a controversial topic, sewage sludge, reclaimed water and microalgae are already being used in agriculture to promote the growth of several crops both in the greenhouse and in the field (Eid et al., 2022; Khaskhoussy et al., 2022; Koutroubas et al., 2023; Mückschel et al., 2023). The most common studied plants were cereals such as wheat and rice that are C_3_ plants, therefore there is a great need to investigate other crops such as C_4_ plants which have different nutritional requirements.

Eid, E. M., Shaltout, K. H., Alamri, S. A., Alrumman, S. A., Taher, M. A., El-Bebany, A. F., ... & Nessem, A. A. (2022). Planned Application of Sewage Sludge Recirculates Nutrients to Agricultural Soil and Improves Growth of Okra (*Abelmoschus esculentus* (L.) Moench) Plants. **Sustainability,** *14*(2), 740.

Khaskhoussy, K., Kahlaoui, B., Misle, E., & Hachicha, M. (2022). Impact of irrigation with treated wastewater on physical-chemical properties of two soil types and corn plant (Zea mays). ***Journal of Soil Science and Plant Nutrition*,** *22*(2), 1377-1393.

Koutroubas, S.D.; Antoniadis, V.; Damalas, C.A. Sideris Fotiadis, S. (2023). Municipal Sewage Sludge Effects on Maize Yield, Nitrogen Use Efficiency, and Soil Properties. **Journal of Soil Science and Plant Nutrition *23*(1), 1209-1221.**

Mückschel, F., Ollo, E., Glaeser, S. P., Düring, R., Yan, F., Velten, H., ... & Frei, M. (2023). Nitrogen use efficiency of microalgae application in wheat compared to mineral fertilizer. **Journal of Plant Nutrition and Soil Science.**

2. The statistical analysis is not quite clear. There is need to mention how data has been statistically analysed separate section.

***Reply:*** The Statistical analysis were clarified in the manuscript as suggested by the reviewer.

Change in the Section Statistical analysis. The statistical analysis were applied to each group of plants separately.

3. Minor Suggestions: The minor suggestions is mentioned in the attached file

***Reply:*** Corrections in the manuscript were done as suggested by the reviewer. See below.

Other comments from reviewer 1:

The graph neither showing any standard error bars nor any type of statistical letttering etc

In the Figure 1, the standard error was introduced. The letters were not added because the curves are too close.

The table should be self explanatory, the units of parameters are missing

The unit was added in the Table 1 caption.

The lettering of graph bars are not approritae, need to correct

The lettering in the Figure 3 were changed.

Write exact instrument specification, including model number, comapny and city of manufacture etc

The additional information of the equipment was added in the section Gas exchange. measurements and photosynthetic pigments determination: LCpro, Portable Photosynthesis System, ADC Bioscientific Ltd., Hoddesdon, UK.

**Reviewer #2: Blue colour in the manuscript**

**Reviewer #2:** The manuscript titled Biofertilizers from wastewater treatment as a potential source of mineral nutrients for growth of amaranth plants is very interesting. However, the following justifications are much needed for the manuscript:

1. Why amaranth plants? Did the authors try with any other plants?

***Reply:*** Amaranth plants hold significant agronomic importance due to the exceptional nutritional quality found in both their leaves and seeds. As stated in the revised introduction, most of the research in this field has predominantly focused on cereals and other C_3_ plants, less data are available on C_4_ plants. Looking ahead, our objective is to explore other species to establish a connection between the addition of biofertilizers and the resultant plant growth and physiological responses.

2. Mention the physiochemical composition of the wastewater?

***Reply:*** Thank you for your observation. A new section was added in Material and Methods e and the physical-chemical composition of sanitary sewage will be included in the text of the article as a Supplementary Information.

Modification to the text (topic *“Collection of previously treated sewage and biosolid obtention” :) […] The sanitary sewage previously anaerobically treated used for cultivation of microalgae was collected at the STP in the district of Tibiriçá, in Bauru/Sao Paulo (22°13’38.7”S 49°1 2’47.0”W). The anaerobic treatment took place in a anaerobic upflow filter, with a volume of 87.30 m3 and a hydraulic detention time (HDT) of 5.05 h. The sludge was obtained in the third chamber of the baffled anaerobic reactor, which had three chambers with a total volume of 597 L and HDT of 8 h. After collecting the sludge was oven dried at 60°C. Then, it was crushed and ground in a mill to facilitate the absorption by the plants****.*** *In the* Supporting Information*, S1 Table describes the physiochemical composition of the sanitary sewage post-treated by the anaerobic upflow filter (UAF) and the baffled anaerobic reactor (BAR) while the concentrations of heavy metals in the sewage treated by UAF and ABR are presented in S2 Table”. There was no significant difference for most parameters of the two raw anaerobic sewage.”*

Supporting Information; S1 and S2 Tables.

**S1 Table.** Characteristics of anaerobically digested sewage by UAF (Upflow anaerobic Filter) and BAR (baffled anaerobic reactor). Values are mean±SD of 72 and 24 replications for UAF and BAR, respectively. *P* = probability.

| **Parameters** | **UAF**  **Mean±SD** | **BAR**  **Mean±SD** | **Difference in effluent between reactors** |
| --- | --- | --- | --- |
| Dissolved oxygen (mg L^-1^) | 3.9 ± 1.5 | *-* | *P* = 0.004 |
| pH | 7.5 ± 0.5 | 7.5 ± 0.5 | *P* = 0.623 |
| TSS (mg L^-1^) | 265.3 ± 109.9 | 211.1 ± 111.5 | *P* = 0.063 |
| DQO_d_ (mg L^-1^) | 178,6 ± 50,1 | 151.0 ± 29.7 | *P* = 0.197 |
| N-organic (mg L^-1^) | 21.1 ± 2.1 | 24.4 ± 10.6 | *P* = 0.820 |
| N-amoniacal (mg L^-1^) | 90.8 ± 20.1 | 71.8 ± 15.2 | *P* = 0.194 |
| NO_2_ (mg L^-1^) | 0.4 ± 0.6 | 0.2 ± 0.3 | *P* = 0.383 |
| NO_3_ (mg L^-1^) | 12.4 ± 7.9 | 12.8 ± 7.4 | *P* = 0.544 |
| Total phosphorus (mg L^-1^) | 11.3 ± 2.0 | 9.9 ± 2.0 | *P* = 0.016 |
| Phosphate (mg L^-1^) | 27.0 ± 5.5 | 25.2 ± 4.8 | *P* =0.527 |
| Alkalinity (mg L^-1^) | 195.9 ± 15.1 | 196.2 ± 23.9 | *P* = 0.312 |
| Turbidity (NTU) | 220.3 ± 64.1 | 207.0 ± 91.7 | *P* = 0.038 |

**TSS - total suspended solids; DQO_d_ - dissolved chemical oxygen demand**

**S2 Table**. Concentration of heavy metals of digested sewage after UAF (Upflow anaerobic Filter) and after BAR (baffled anaerobic reactor).

| **Parameters**  **(mg L^-1^)** | *UAF* | *ABR* |
| --- | --- | --- |
| **Al** | 0.635 | 0.402 |
| **As** | 0.465 | 0.110 |
| **Ba** | 0.180 | 0.121 |
| **Ca** | 17.984 | 15.347 |
| **Cd** | 0.013 | 0.006 |
| **Co** | 0.020 | 0.009 |
| **Cr** | 0.019 | 0.006 |
| **Cu** | 0.060 | 0.018 |
| **Fe** | 0.238 | 0.276 |
| **K** | 27.889 | 5.562 |
| **Mg** | 10.408 | 3.884 |
| **Mn** | 0.134 | 0.065 |
| **Na** | 26.140 | 48.773 |
| **Ni** | 0.033 | 0.010 |
| **Pb** | 0.039 | 0.070 |
| **Se** | 2.230 | 0.148 |
| **Zn** | 0.307 | 0.032 |

3. Was heavy metal analysis done for the wastewater?

***Reply: See above for the analyses of heavy metals.***

4. The authors need to justify the reason for utilization of wastewater and address the constraint with the safety regulation related to the usage of wastewater.

***Reply:*** The recovery of N and P from sanitary sewage presents a significant opportunity, potentially meeting up to 30% of the nitrogen demand and 22% of the phosphorus demand in agriculture (Verstraete et al., 2009; Mihelcic et al., 2011; Suleiman et al., 2020). Additionally, the reuse of previously treated sewage is a valuable endeavour. However, conventional sewage treatment methods often fall short in efficiently removing these essential nutrients. A promising solution involves tertiary treatment with microalgae, which proves to be an effective strategy. This approach allows for the extraction of valuable nutrients from microalgae biomass. An advantage of anaerobic treatment is its ability to partially remove approximately 70% of organic matter while preserving nutrient content (Zeeman et al., 2008). Furthermore, microalgae can be cultivated in the pre-treated effluent, serving as a tertiary treatment method, and enabling the recovery of both micro and macronutrients from the effluent (Suleiman et al., 2020). In countries like Brazil, which heavily rely on fertilizer imports, adopting such an approach reduces the necessity to exploit mineral resources and produce chemical fertilizers (Withers et al., 2018). Moreover, the effluent treated with microalgae can be safely used in agricultural irrigation, promoting water reuse, thereby contributing to the conservation of precious water resources, and reducing the dependency on potable water (Lyu et al., 2022).

In Brazil, the reuse of treated wastewater for agricultural purposes is governed by CONAMA Resolution No. 357, along with complementary resolutions, which establish the categories of effluents and their designated uses (Brazil, 2005). Furthermore, apart from Brazilian regulations, there are guidelines from the World Health Organization (WHO) that provide safety parameters for using wastewater in irrigation. When it comes to the utilization of sewage sludge for agricultural purposes, it is regulated by CONAMA Resolution No. 498. The primary safety parameter in this context is the analysis of thermotolerant coliforms, specifically Escherichia coli, with a maximum allowable limit of 10^3^ *Escherichia coli* per gram of total solids for biosolid application in soil. Additionally, concentrations of heavy metals are monitored (Brazil, 2020). In the case of using microalgae biomass cultivated in wastewater, there are currently no specific regulations. However, it could be suggested that the same standards outlined in CONAMA Resolution No. 498 be applied to assess the safety of this biomass. It's worth noting that, in addition to the inherent safety measures like the drying process that sewage sludge undergoes, which has a disinfecting effect, microalgae also contribute to disinfection of the environment in complementary ways. This leads to a substantial reduction in pathogens, as demonstrated in a study by Ruas et al. (2021).Parte superior do formulário

Parte inferior do formulário

Parte superior do formulário

*Following the reviewer's notes, we will highlight these points throughout the text and mention the regulations on the use of sanitary sewage.*

*Brazilian regulation on the use of wastewater – CONAMA 357 and 430; CONAMA 397*

*BRASIL. Ministério do Meio Ambiente (MMA). Conselho Nacional do Meio Ambiente (CONAMA). Resolução CONAMA Nº 357 de 17 de março de 2005. Dispõe sobre a classificação dos corpos de água e diretrizes ambientais para o seu enquadramento, bem como estabelece as condições e padrões de lançamento de efluentes, e dá outras providências. 2005.*

*BRASIL. Ministério do Meio Ambiente (MMA). Conselho Nacional do Meio Ambiente (CONAMA). Resolução CONAMA Nº 498 de 19 de agosto de 2020. Define critérios e procedimentos para produção e aplicação de biossólido em solos, e dá outras providências. 2020.*

*LYU, Sidan et al. Effects of reclaimed wastewater irrigation on soil-crop systems in China: A review. Science of The Total Environment, v. 813, p. 152531, 2022.*

*Mihelcic JR, Fry LM, Shaw R. Global potential of phosphorus recovery from human urine and feces. Chemosphere. 2011;84(6):832–839.*

*Ruas G, Serejo M, Farias S, Scarcelli P, Boncz M. Removal of pathogens from domestic wastewater by microalgal-bacterial systems under different cultivation conditions. International Journal of Environmental Science and Technology. 2021; p. 1–12.*

*Suleiman AKA, Lourenço KS, Clark C, Luz RL, da Silva GHR, Vet LE. From toilet to agriculture: Fertilization with microalgal biomass from wastewater impacts the soil and rhizosphere active microbiomes, greenhouse gas emissions and plant growth. Resources, Conservation and Recycling. 2020;161:104924.*

*Verstraete W, Van de Caveye P, Diamantis V. Maximum use of resources present in domestic “used water”. Bioresource technology. 2009;100(23):5537–5545.*

*WITHERS, P. J. et al. Transitions to sustainable management of phosphorus in Brazilian agriculture. Scientific reports, v. 8, n. 1, p. 2537, 2018. DOI: 10.1038/s41598-018-20887-z*

*WORLD HEALTH ORGANIZATION. WHO guidelines for the safe use of wastewater excreta and greywater. World Health Organization, 2006.*

*Zeeman G, Kujawa K, De Mes T, Hernandez L, De Graaff M, Abu-Ghunmi L, et al. Anaerobic treatment as a core technology for energy, nutrients, and water recovery from source-separated domestic waste (water). Water Science and Technology. 2008;57(8):1207–1212.*

*Modification to the text (topic “Introduction” line 22): “However, when subjected to additional treatments in order to reduce the water content and/or to stabilize the organic matter and remove pathogens [11, and references therein], it results in a biosolid that is easy to handle for disposal or use as a biofertilizer in agriculture because it has a large amount of nutrients and organic matter [12],* ***and additionally, these drying and disinfection processes guarantee the suitability of the biosolids to safety regulations (Brasil, 2020).”***

*Modification to the text (topic “Introduction” line 55): [...]In addition to its value as a source of water, reclaimed water has also been shown to be efficient in increasing the productivity of several crops of economic interest due to the presence of essential nutrients necessary for plant growth despite the presence of some metals [31–33]. In Brazil, reclaimed water must meet the standards established in CONAMA resolution nº 357. Specific requirements are determined based on the type of effluent and the intended crop for which the reused water will be used (CONAMA, 2005). According to the safety guidelines of the World Health Organization, the established parameter concerns the maximum permitted value of E. coli, which must be between 10^3^ and 10^4^, depending on the culture, whether it is consumed raw and whether there is direct contact with the edible part with the soil (WHO, 2006).*

Parte superior do formulário

5. The abstract and conclusion need to be improvised as they are too generalised and no information on the key experimental findings of the research.

***Reply:*** The abstract and conclusion were changed as requested by the Reviewer.

**New abstract**

Exploring alternative fertilizers is crucial in agriculture due to the cost and environmental impact of inorganic options. This study investigated the potential of biofertilizers derived from sewage treatment on the growth and physiology of *Amaranthus cruentus* plants. Various treatments were compared, including control treatments with inorganic fertilizer and treatments with biofertilizers composed of microalgae, biosolid and reclaimed water. The following traits were investigated: photosynthetic pigments, gas exchange, growth, and leaf nutrient concentrations. The results show that the concentration of N, P, Cu, Fe, Zn and Na, in the dry microalgae and in the biosolid, were quite high for the needs of the plants. The wet microalgae presented high concentration of Cu, Fe and Zn while reclaimed water contained high concentration of N, K, Ca and S. Na and Zn increased in the leaf of plants treated with dry microalgae and biosolid, respectively. At the beginning of the flowering phase, total chlorophyll and carotenoids contents were lower for plants grown with wet microalgae while for plants grown with higher doses of biosolid or reclaimed water total chlorophyll was increased, and carotenoids were not affected. Lower photosynthetic pigments under wet microalgae resulted in lower photosynthetic rates. On the other hand, amendments with dry microalgae and biosolid increased photosynthetic rates with the biosolid being the most effective. Higher applications of biosolid, wet and dry microalgae produced a considerable increase in shoot biomass of amaranth, with the dry microalgae being the most effective. Additionally, reclaimed water obtained after tertiary treatment of sewage with microalgae and biosolid applied alone showed promising effects on plant growth. Overall, these findings suggest that organic fertilizers derived from sewage treatment have the potential to enhance plant growth and contribute to sustainable agricultural practices.

**New conclusion**

This study investigated the potential of organic fertilizers from domestic sewage treatment, such as biosolids, wet and dry microalgae and the reclaimed water to enhance the biomass of aerial parts of amaranth plants. The characterization of the organic fertilizer used in this experiment shows that their nutrient content is unbalanced in relation to plant needs. Despite the nutrient deficiencies observed in some treatments, the application of larger doses of these products significantly increased the aerial biomass production, with dry microalgae being the most effective. The partitioning of dry matter in the leaves was greater than in the stem probably due to N content in the biofertilizers used in this study. While the treatments receiving exclusively reclaimed water or sludge did not surpass the 20% control treatment, they demonstrated the potential for biomass production with the available nutrients. Amendments with dry microalgae and biosolid increased photosynthetic rates with the biosolid being the most effective due to the increase in total chlorophyll content. To optimize initial growth, further investigations are needed to determine the appropriate doses and application rates of these products. Additionally, the high concentrations of micronutrients such as Cu, Fe, Zn, and Na in the biosolids and microalgae biomass require careful consideration to avoid potential stress on plants. Overall, our findings suggest that organic fertilizers from domestic sewage treatment can offer a sustainable solution for nutrient cycling by returning them to the soil and promoting plant growth in nutrient-poor soils.

**Reviewer #3: Violet colour in the manuscript**

**Reviewer #3:** The manuscript evaluates the sewage-grown microalgae as potential biofertilizer. The concept and results are provided in meaningful manner which will help in the real-time implementation of proposed biofertilizer. Various aspects of biofertilizers had been evaluated by authors which is good thing, however few recommendations are given below for the improvement of manuscript, prior to its acceptance for PLOS One Journal.

• It would be interesting to present the quantitative biochemical composition of Amaranthus in the introduction section.

***Reply:*** As suggested by the reviewer, in the Introduction some references containing the biochemical composition of amaranth have been added.

**Introduction: Last paragraph.** Amaranthus is a C_4_ plant of great agronomic importance because of its high nutritional and nutraceutical values which provide health benefits. Manyelo et al. (2022) reported higher Ca, P, Mg and K contents (2771.07, 5024.56, 3501.36 and 5101.99 mg/kg, respectively) in the grain and significantly higher trace mineral content for Cu, Mn, Zn and Fe (5.95, 23.71, 49.97 and 147.01 mg/kg, respectively). In addition, secondary metabolites, specifically anthocyanins, were shown to be abundant in grains (35.92 mg/kg), which reinforces its potential in increasing the immunity. Amaranth grains also have high content of starch (65-75%), proteins (15.4-16%), lipids (6.98-7.22%) and several vitamins (Malik et al., 2023), besides being gluten free, making it important for celiac patients (Alemayehu et al., 2015).

References

Manyelo, T. G., Sebola, N. A., Hassan, Z. M., Ng’ambi, J. W., Weeks, W. J., & Mabelebele, M. (2022). Chemical composition and metabolomic analysis of *Amaranthus cruentus* grains harvested at different stages. **Molecules,** *27*(3), 623.

Malik, M., Sindhu, R., Dhull, S. B., Bou-Mitri, C., Singh, Y., Panwar, S., & Khatkar, B. S. (2023). Nutritional Composition, Functionality, and Processing Technologies for Amaranth. **Journal of Food Processing and Preservation**, *2023*.

• What was the reason of blocking light radiations during algal growth? Generally, higher light is associated with better nutrient removal and biomass growth.

***Reply:*** High light intensities can inhibit the photosynthetic activity of microalgae, and consequently reduce the ability to remove nutrients from the system (Assemany et al., 2015). The control of light intensity with shading of 50% of the lighted area was based on previous studies carried out by Ruas et al. (2022), where it was demonstrated that the use of shading favours the diversity of the reactors' microalgae community and prevents photoinhibition, without harming the ability to eliminate pathogens.

*ASSEMANY, Paula Peixoto et al. Algae/bacteria consortium in high rate ponds: influence of solar radiation on the phytoplankton community.* ***Ecological Engineering****, v. 77, p. 154-162, 2015.*

*RUAS, Graziele et al. CO2 Addition and Semicontinuous Feed Regime in Shaded HRAP—Pathogen Removal Performance.* ***Water****, v. 14, n. 24, p. 4047, 2022.*

*Modification to the text (topic “microalgae cultivation”): The cultivation of microalgae took place at room temperature under a shading fabric that blocked 50% of natural light radiation (Solpack), which according to Ruas et al. (2022) is a method of preventing photoinhibition and favoring the diversity of the reactor community without compromising the disinfection capacity.*

• Please provide the details of the algal strain which is used in this experiment.

***Ans:*** *Information about the strain of microalgae used was included in the text of the article.*

*Modification to the text (topic “microalgae cultivation”): [...]The microalgae cultivation took place between the months of January and April (summer/fall), with an average temperature of 280C, and the average light intensity of 919.48 μmol m^−2^s^−1^. In the current investigation, the most prevalent genera of microalgae identified were colonial cyanobacteria, specifically Aphanotece sp. and Aphanocapsa sp., along with filamentous algal species. Following these, Chlorella sp., Scenedesmus sp., and Chlamydomonas sp. were also present. The microalgae utilized in this study were sourced through a bioprospecting process, which enabled the selection of species naturally occurring in sewage. Consequently, these microalgae were already well-adapted to the culture medium, facilitating their growth and development throughout the experimental process (Wilkie et al., 2011).*

*WILKIE, Ann C.; EDMUNDSON, Scott J.; DUNCAN, James G. Indigenous algae for local bioresource production: Phycoprospecting.* ***Energy for sustainable development****, v. 15, n. 4, p. 365-371, 2011.*

• Usually, algal growth is compromised in the presence of sludge or suspended solids. What was the behaviour in present study? Which technique was used to obtain algal biomass in murky wastewater?

***Reply:*** The sewage used as a growth medium for microalgae underwent prior treatment in an upflow anaerobic filter. At the outlet of this filter, the concentration of total solids measured 265.3 ± 109.9 mg L^-1^, while turbidity registered at 220.3 ± 64.1 NTU (see S1 Table in supporting information). Anaerobic treatment has the important characteristic of removing organic matter from sewage while preserves the nutrients present. This pre-treated sewage, therefore, serves as a valuable source for recovering these essential nutrients, as highlighted in studies by SLOMPO et al. (2019) and ZEEMAN et al. (2008). Due to the removal of organic matter during this process, the average concentrations of suspended solids and turbidity did not hinder the production of microalgae biomass, which reached 280 mg L^-1^, as indicated by research conducted by De Souza Leite et al. (2019) and Shen et al. (2017).

Parte superior do formulário

*DE SOUZA LEITE, Luan; HOFFMANN, Maria Teresa; DANIEL, Luiz Antonio. Microalgae cultivation for municipal and piggery wastewater treatment in Brazil. Journal of Water Process Engineering, v. 31, p. 100821, 2019.*

*SHEN, Liang et al. Assessment upon heterotrophic microalgae screened from wastewater microbiota for concurrent pollutants removal and biofuel production. Bioresource technology, v. 245, p. 386-393, 2017.*

*SLOMPO, N. D. M. et al. Black water treatment by an upflow anaerobic sludge blanket (UASB) reactor: a pilot study. Water Science and Technology, v. 80, n. 8, p. 1505-1511, 2019. DOI: 10.2166/wst.2019.402.*

*ZEEMAN, G. et al. Anaerobic treatment as a core technology for energy, nutrients and water recovery from source-separated domestic waste (water). Water Science and Technology, v. 57, n. 8, p. 1207-1212, 2008. DOI: 10.2166/wst.2008.101*

• Do the authors perform the safety assessment to evaluate the impact of tannin on biofertilizer, as this information is of importance for real-time application of proposed biofertilizer.

***Reply:*** No studies were conducted to verify the safety of using tannin with biofertilizer, and this is certainly a gap that should be explored in future studies. While there are some articles highlighting the effectiveness of tannin in separation of microalgae, with some studies indicating that tannin does not harm microalgae cells (Teixeira et al., 2022; Yang et al., 2021; Hou et al., 2018), it remains essential to investigate the specific impacts of tannin in biofertilizers. This includes an exploration of whether tannin interacts with nutrients, potentially affecting their availability, or if there are any consequences for the species cultivated using such biofertilizers. Thorough investigation in this area is crucial to ensure the safe and effective use of tannin in biofertilizer applications.

Parte superior do formulário

*TEIXEIRA, Mariana Souza et al. Tannin-based coagulant for harvesting microalgae cultivated in wastewater: Efficiency, floc morphology and products characterization.* ***Science of The Total Environment****, v. 807, p. 150776, 2022.*

*YANG, Zijun; HOU, Jun; MIAO, Lingzhan. Harvesting freshwater microalgae with natural polymer flocculants.* ***Algal Research****, v. 57, p. 102358, 2021.*

*HOU, Jun et al. Changes in Microcystis aeruginosa cell integrity and variation in microcystin-LR and proteins during Tanfloc flocculation and floc storage.* ***Science of the Total Environment****, v. 626, p. 264-273, 2018.*

• Authors mentioned that 70% LA promotes the plant growth, then why this condition was not selected for the biofertilizer applications?

***Reply:*** In our experimental setup, we utilized two control groups. The first control featured a 20% Long Ashton solution (LA), simulating conditions of poor soil fertility. The second control, comprising a 70% LA solution, represented nutrient-rich soil conditions. It's worth noting that this second control already constituted an optimal environment for amaranth growth, a finding observed in prior experiments. Consequently, no additional nutrient supplementation was necessary for this group. The purpose of including the 70% LA control was to facilitate a comparison, allowing us to assess the extent to which the application of biofertilizers alongside the 20% LA solution stimulated plant growth when contrasted with this already fertile condition.

• Please provide the full form of abbreviation at the first mention of term.

***Reply:*** Full form of abbreviations were added in the text.

• Why authors didn’t check the combination of reclaimed water and algae, as It would be interesting to evaluate their impact.

***Reply:*** This is a particularly important suggestion. In the present project, the aim was to text the effects of microalgae (dry and wet) and the reclaimed water separately. In a future project, we can introduce this suggestion.

*We hope that these modifications will comply with the request of the reviewers and the editor. Please do not hesitate to contact us at your convenience if you need further information.*

| *Elisa Teófilo Ferreira* | *Sarah Corrêa Barrochelo* | *Sarah de Paula de Melo* |
| --- | --- | --- |
| *Thainá Araujo* | *Augusto Cesar Coelho Xavier* | *Gustavo Henrique Ribeiro da Silva* |
| *Inês Cechin* |  |  |
